# Supplementary material for: Powerful eQTL mapping through low-coverage RNA sequencing
Source: HGG Adv. 2022 Apr 2;3(3):100103. doi: 10.1016/j.xhgg.2022.100103 (PMC9062329; doi:10.1016/j.xhgg.2022.100103)
Supplement: Document S1. Figures S1–S14 and Tables S1–S3 [file mmc1.pdf]

HGGA, Volume *3*

Supplemental information

Powerful eQTL mapping through low-coverage RNA sequencing

Tommer Schwarz, Toni Boltz, Kangcheng Hou, Merel Bot, Chenda Duan, Loes Olde Loohuis, Marco P. Boks, René S. Kahn, Roel A. Ophoff, and Bogdan Pasaniuc

SUPPLEMENTARY FIGURES

S1A:

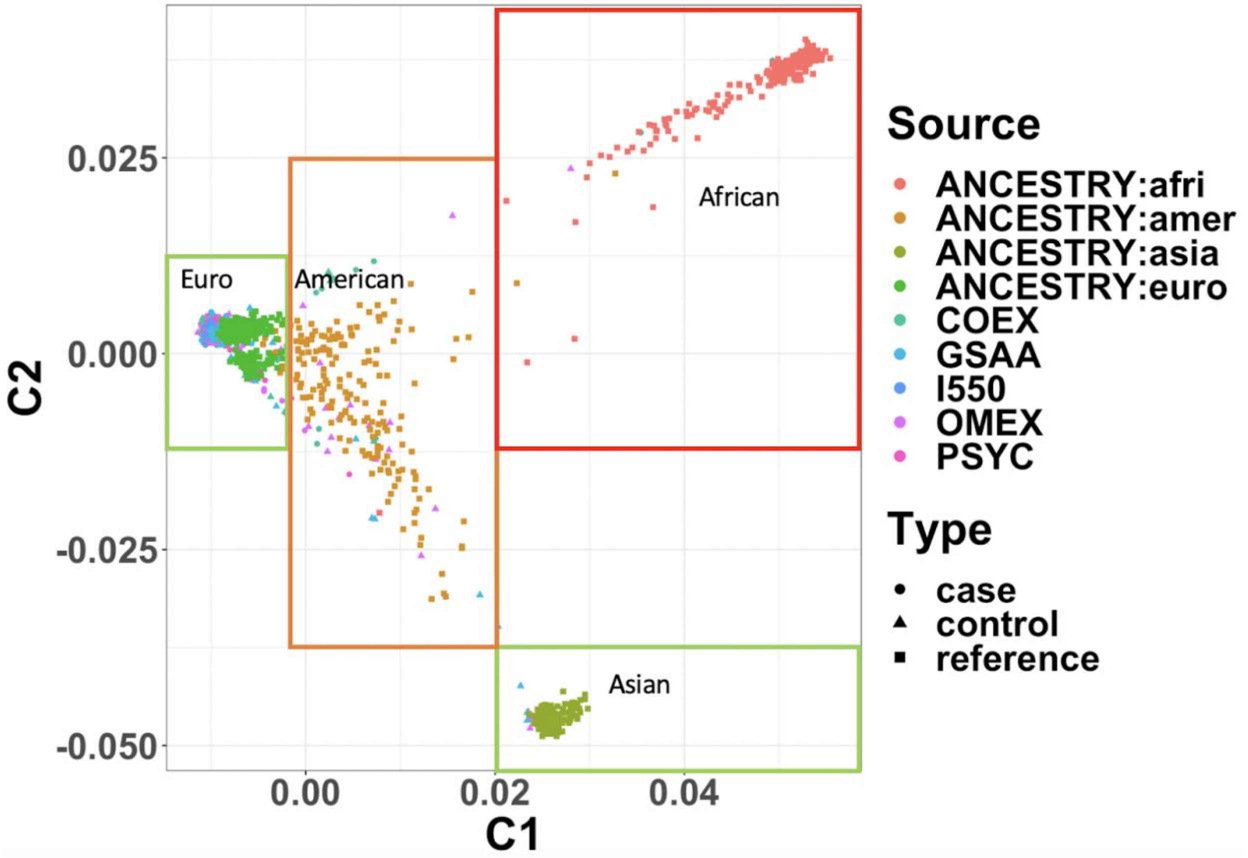

S1B:

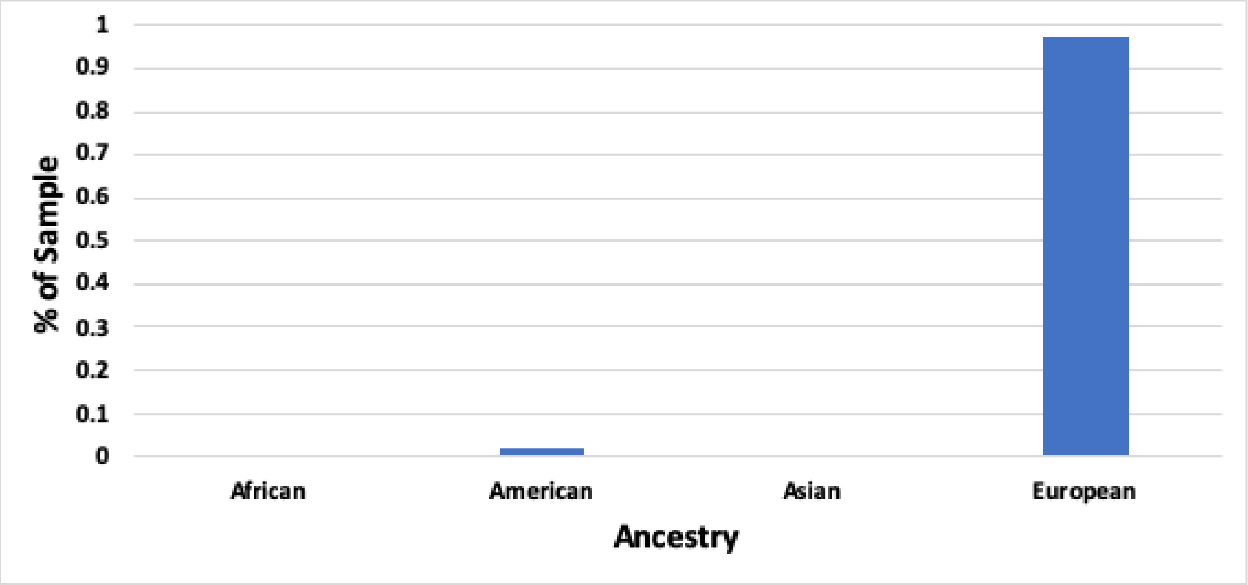

S1C:

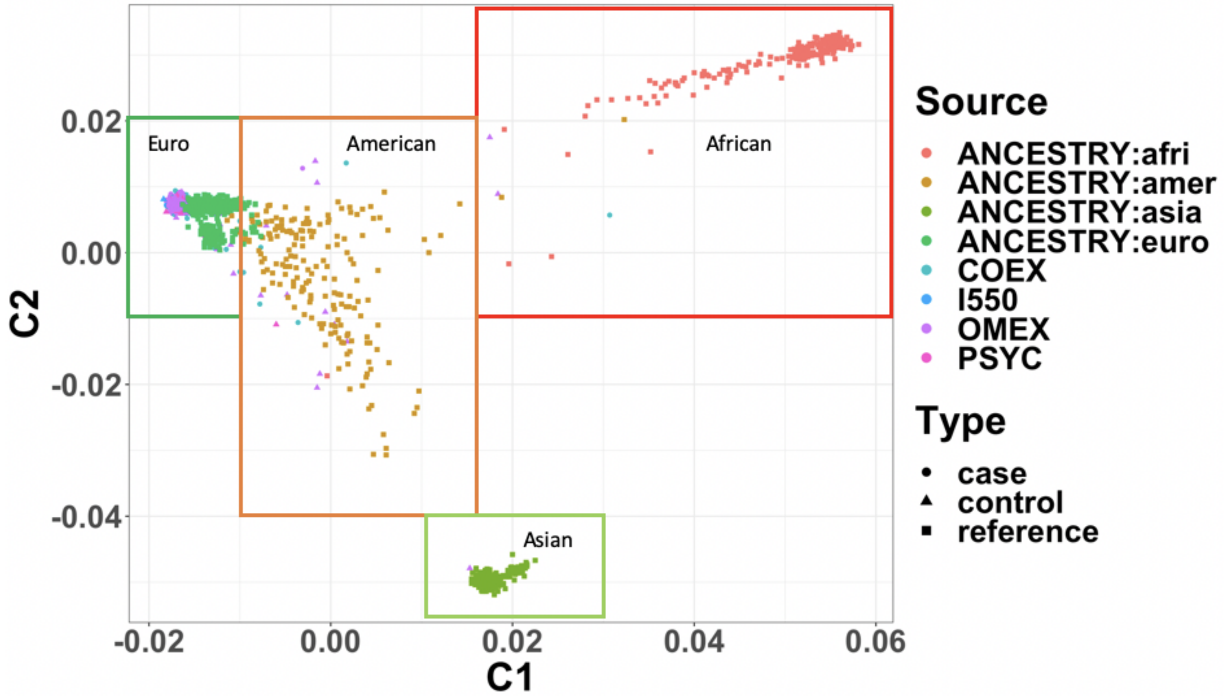

S1D:

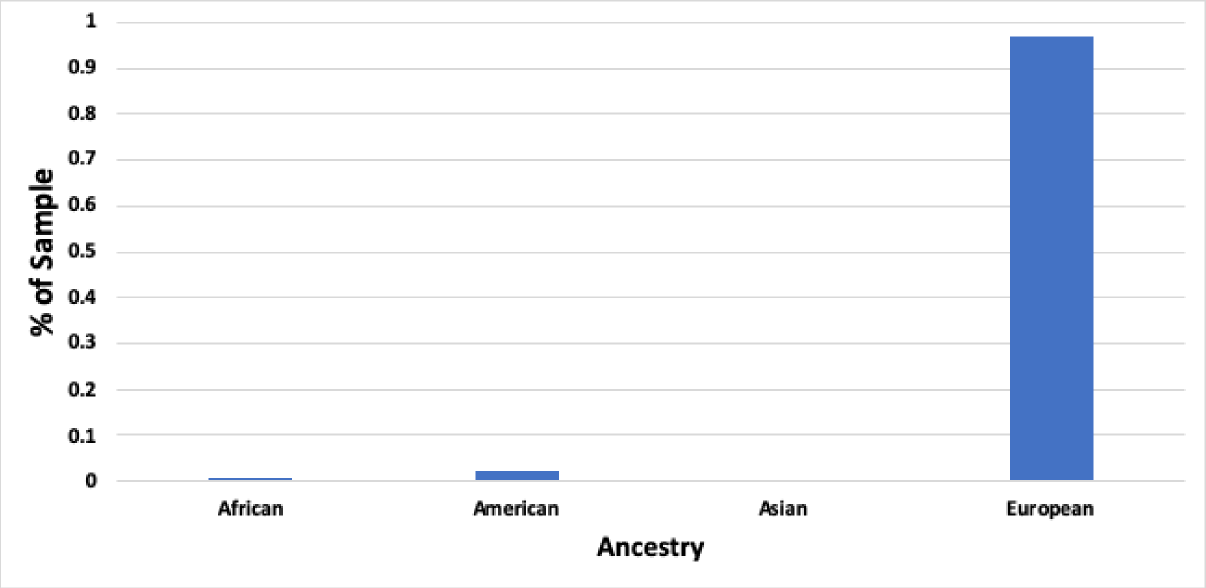

Figure S1: *Distribution of ancestry among samples.* **(S1A)** Genotype PC1 and PC2 are projected onto PCs from 1000 Genomes Project. Points labeled with "ANCESTRY" are from 1000 Genomes Project, remaining points designate the specific genotyping platform used in our cohort. Boxes are drawn around the centers to show where samples from the n = 2000 / 5.9M reads/sample cohort lie. **(S1B)** A barplot showing the distribution of ancestry observed in the n = 2000 / 5.9M reads/sample cohort, according to the MDS plot. Note that only the 1963 samples that pass genotype QC thresholds are included here. Exact numbers of samples per ancestry group are: African - 4, American - 34, Asian - 9, European – 1916. **(S1C)**: Genotype PC1 and PC2 are projected onto PCs from 1000 Genomes Project. Points labeled with "ANCESTRY" are from 1000

Genomes Project, remaining points designate the specific genotyping platform used in our cohort. Boxes are drawn around the centers to show where samples from the n = 759 / 13.9M reads/sample cohort lie. **(S1D)** A barplot showing the distribution of ancestry observed in the n = 759 / 13.9M reads/sample cohort, according to the MDS plot. Exact numbers of samples per ancestry group are: African - 4, American - 19, Asian - 1, European – 735.

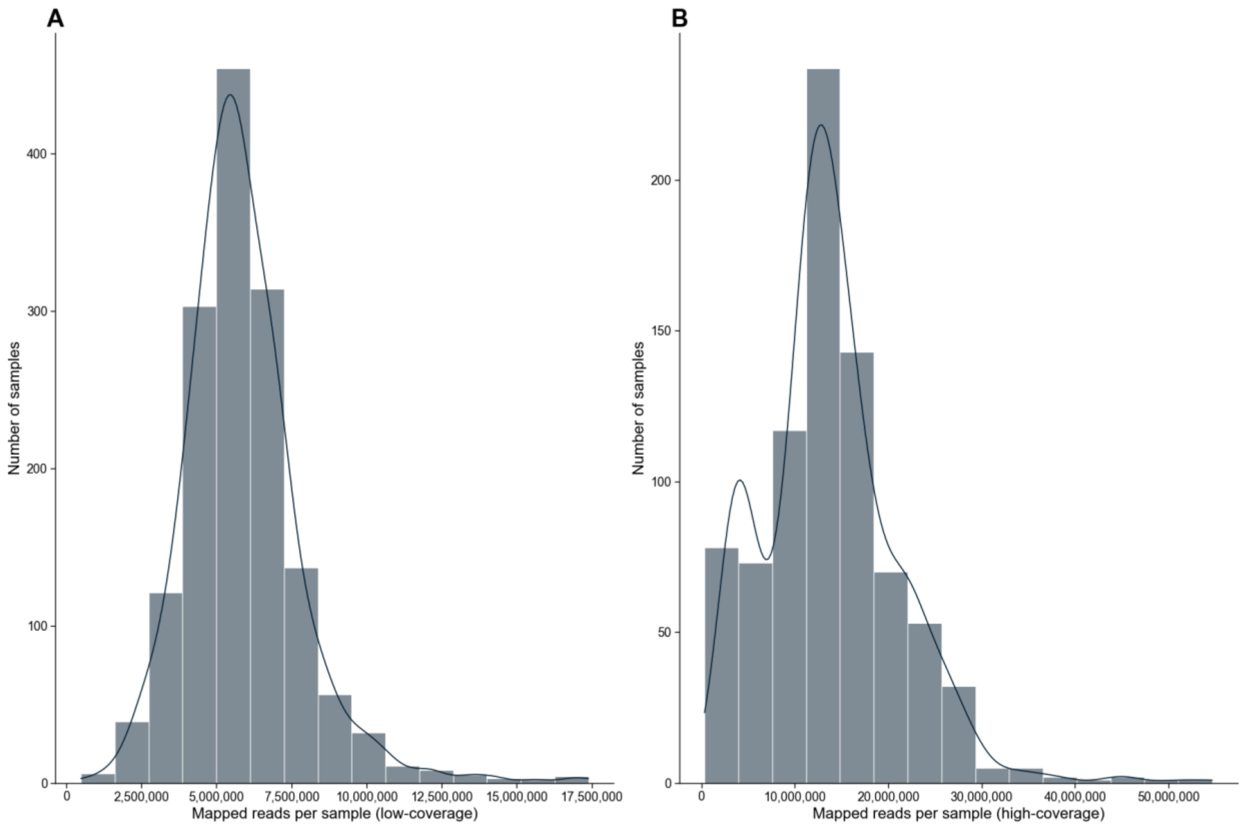

Figure S2: *Number of pseudoaligned reads per sample for low-coverage and high-coverage experiments*. **(S2A)** In real data, a histogram showing the number of reads mapped to genes (or in kallisto terms: number of reads for which transcriptome successfully mapped), per sample. **(S2B)** In real data, a histogram showing the number of reads mapped to genes (or in kallisto terms: number of reads for which transcriptome successfully mapped), per sample.

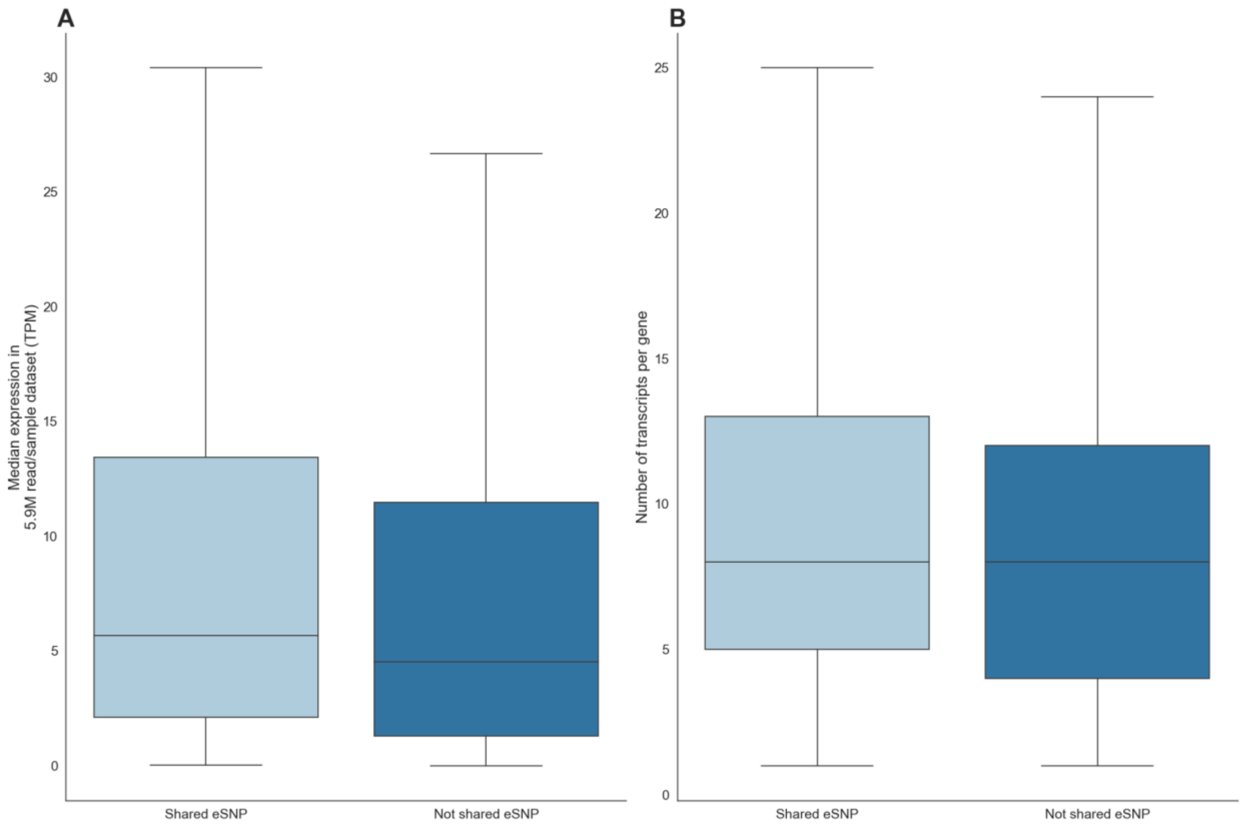

Figure S3: *Characteristics of eGenes that do/do not share the same eSNP between lower-coverage and moderate-coverage RNA-Seq.* **(S3A)** eGenes with the same eSNP have an average expression of 15.6 TPM (sd = 51.1) while eGenes that do not share the same eSNP have an expression of 14.2 TPM (sd = 40.8), (p = 0.03). (**S3B)** The average number of isoforms for eGenes that shared an eSNP was 10.0 (sd = 8.0), while it is only 9.6 (sd = 7.9) for those eGenes that do not share an eSNP (p = 0.03).

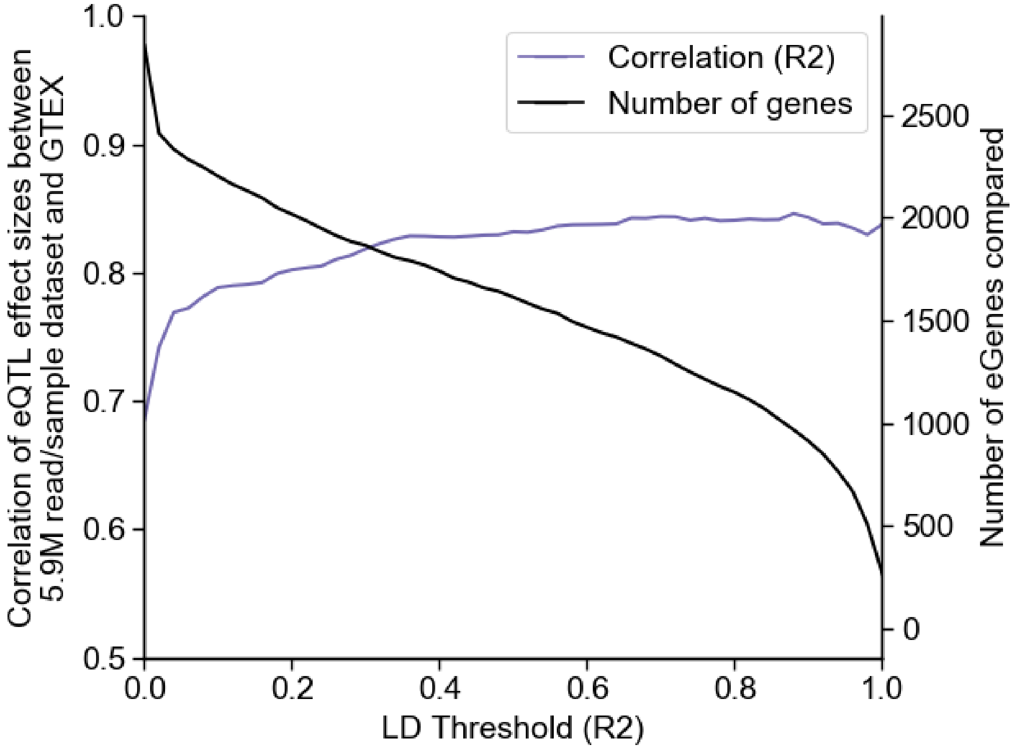

Figure S4: *Correlation of effect sizes between eQTL analyses low-coverage and GTEX with respect to LD-threshold between eSNPs.* **(S4)** On the x-axis, we show the LD threshold used to restrict our comparisons of effect sizes between eQTLs found in the low-coverage dataset and GTEX. Only eGenes with the same eSNP, or eSNPs with an LD above the threshold are used. On the left-hand y-axis, we show the correlation of the effect sizes. On the right-hand y-axis, we show the number of eGenes compared under the given LD threshold.

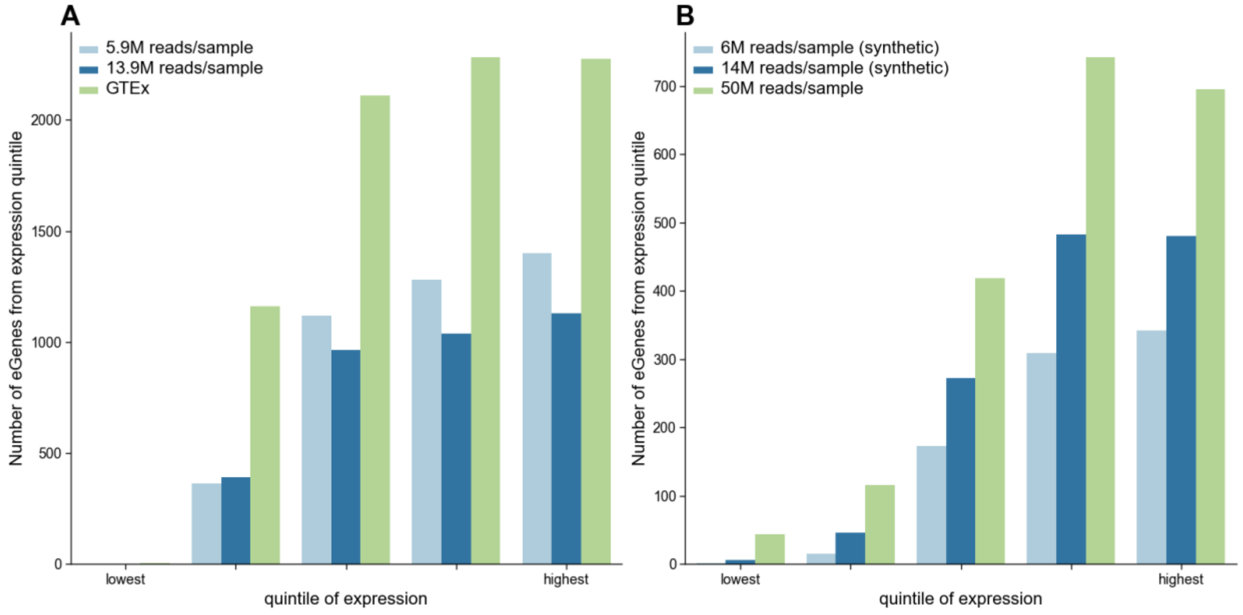

Figure S5: *Number of eGenes per mean expression quintile across datasets.* **(S5A)** Stratifying the 19175 protein coding genes reported in GTEx into quintile groups by mean expression, on the x-axis, we show the quintile groups by increasing mean expression. On the y-axis, we show the number of eGenes found in the (1) low-coverage, (2) high-coverage, and (3) GTEx experiments, in each of these quintile groups. **(S5B)** Stratifying the 24206 protein coding genes discovered in the high-coverage fibroblast dataset into quintile groups by mean expression, on the x-axis, we show the quntile groups by increasing mean expression. On the y-axis, we show the number of eGenes found in the (1) synthetic RNA-Seq at 6M reads/sample, (2) synthetic RNA-Seq at 14M reads/sample, and (3) RNA-Seq data at 50M reads/sample, in each of these quintile groups.

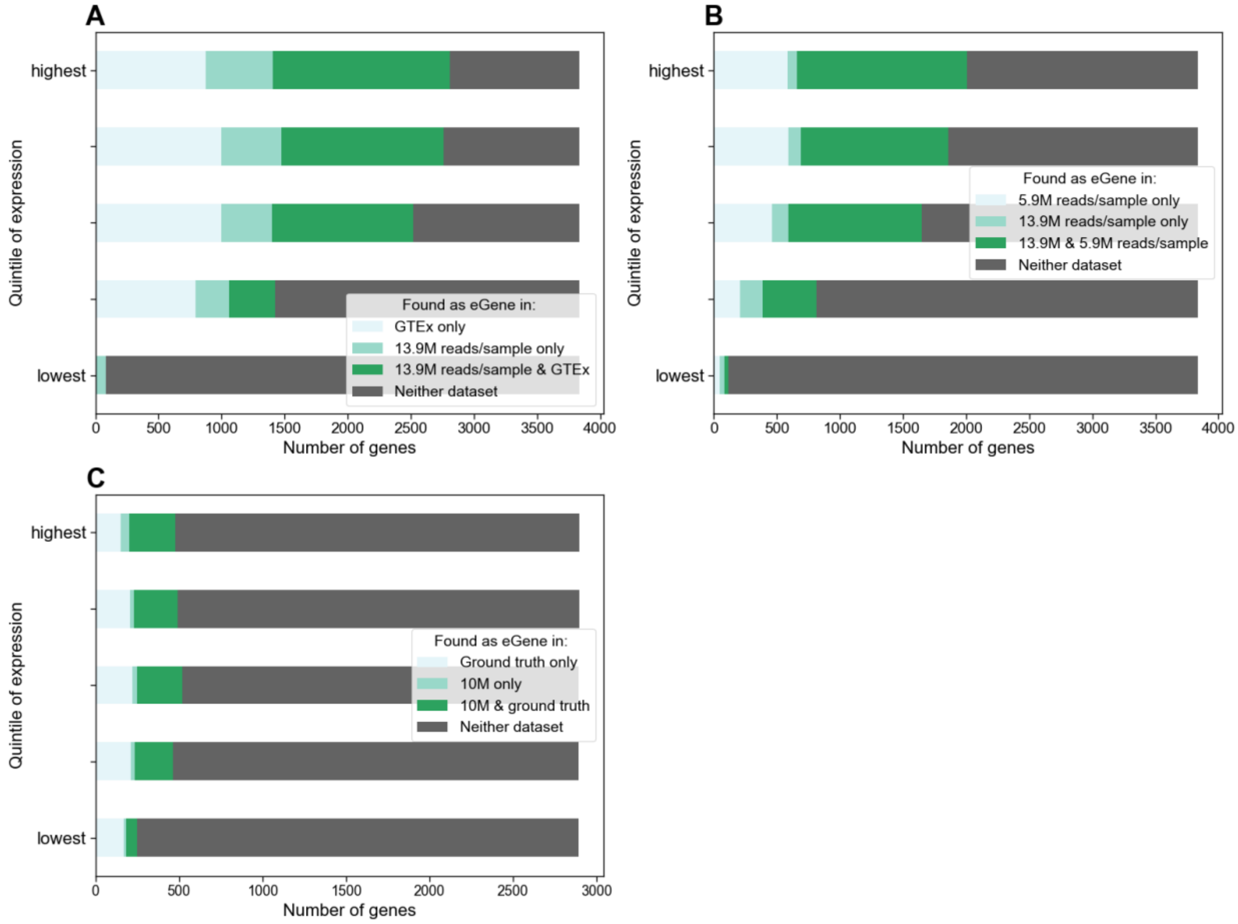

Figure S6: *eGene concordance by quintile in moderate-coverage RNA-Seq and GTEx:* **(S6A):** The overlap in eGenes between moderate-coverage RNA-Seq and GTEX, stratified into quintiles by the mean expression level observed in GTEX. **(S6B):** The overlap in eGenes between moderate-coverage RNA-Seq and lower-coverage RNA-Seq, stratified into quintiles by the mean expression level observed in GTEX. **(S6C):** The overlap in eGenes between high-coverage "ground-truth" RNA-Seq and a 10M read/sample synthetic dataset, stratified into quintiles by the mean expression level observed in the high-coverage RNA-Seq

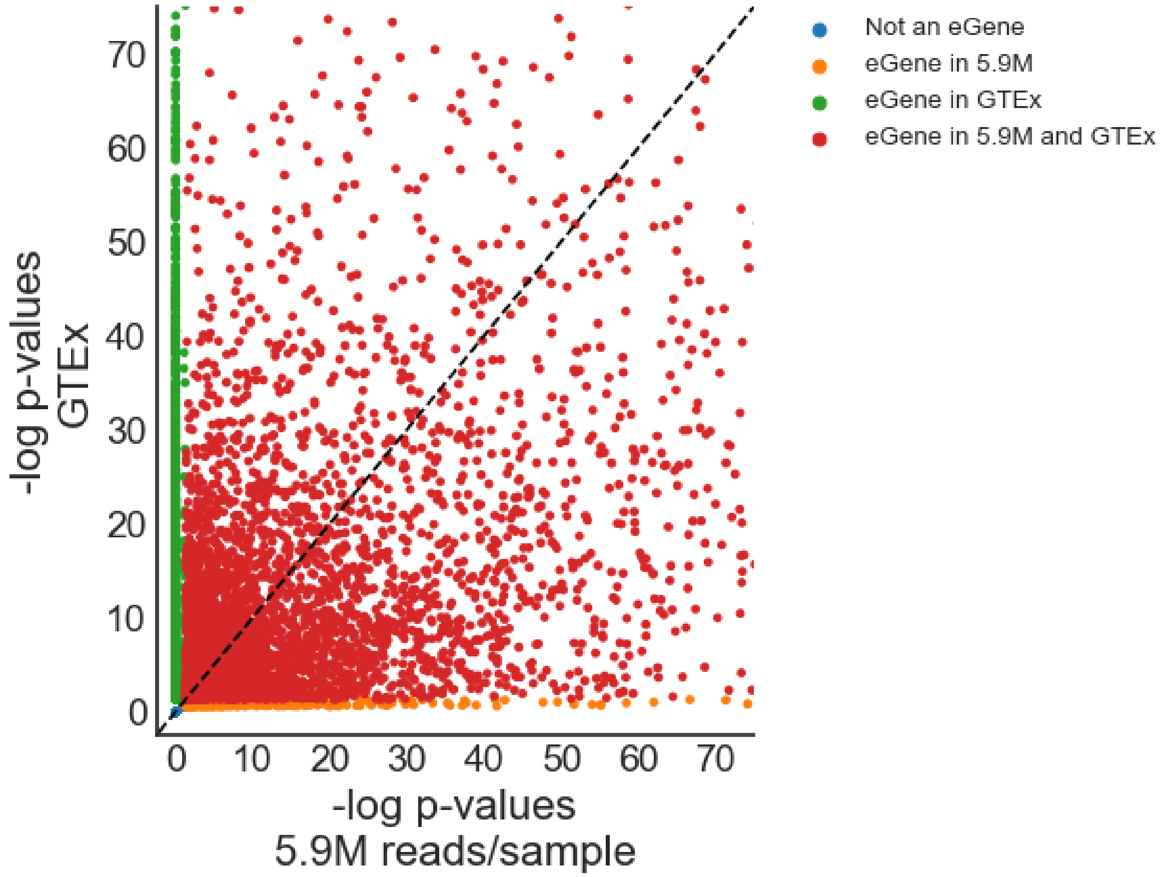

Figure S7: *Real data p-value comparison scatterplot with GTEX*. **(S3A)** Using the 12,496 protein-coding genes included both in GTEX and the low-coverage datasets, on the x-axis, we show the -log p-values for leading SNP eQTL associations in the low-coverage dataset. On the y-axis, we show the -log p-values for leading SNP eQTL associations in the GTEX dataset.

A

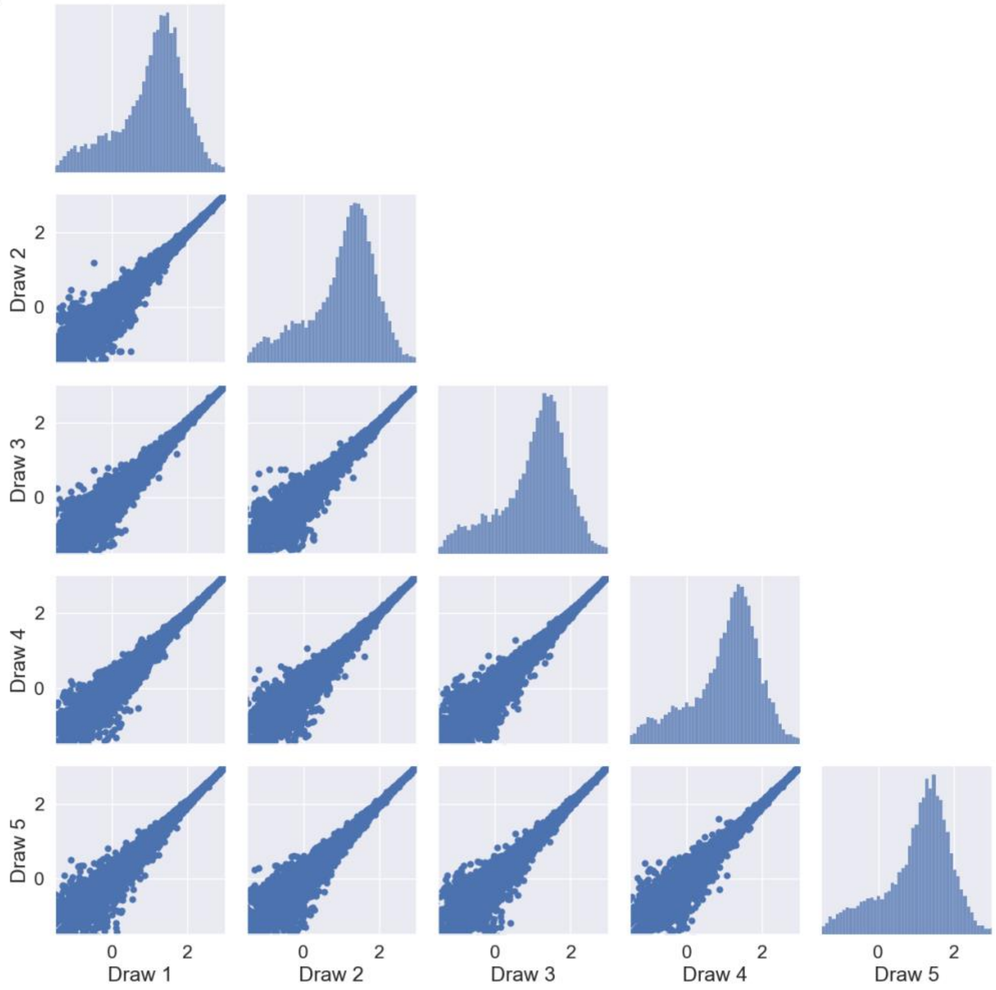

B

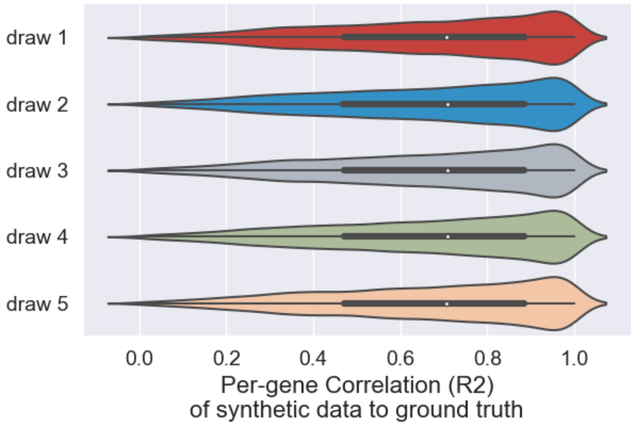

Figure S8: V*ariability in correlations in synthetic data*. **(S8A)** For synthetic data corresponding to one sample, a comparison of estimated log TPM values between five different uniform sampling

draws at 10 million reads/sample, for 14,948 protein-coding genes. **(S8B)** For 14,948 protein-coding genes estimated across five different uniform sampling draws at 10 million reads/sample, we compare the distribution of correlation (R^2) between the estimated expression of the samples and the ground truth gene expression.

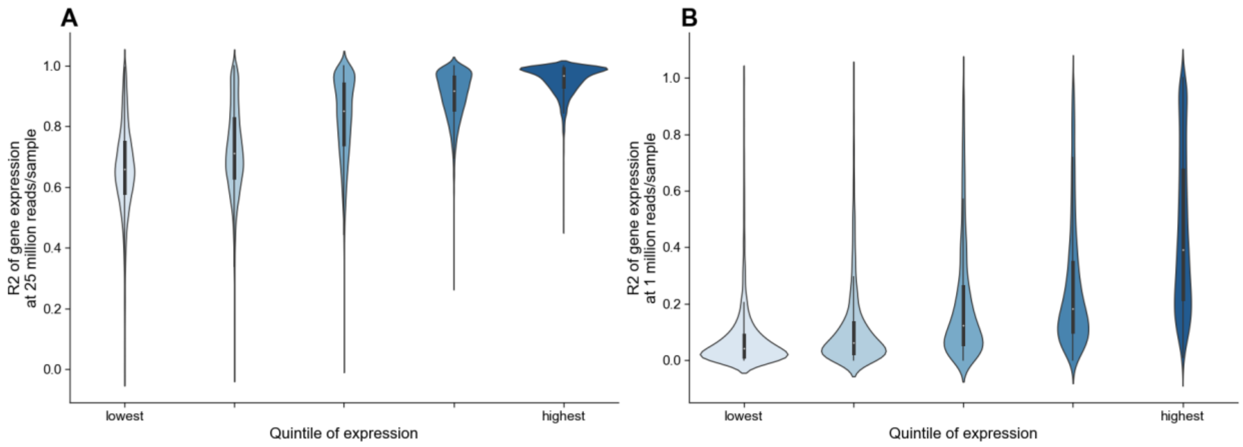

Figure S9: *Variability of correlations as a function of average expression in a given gene* **(S9A)** Gene expression accuracy using data simulated with 1 million reads/sample, as a function of relative gene expression observed in actual RNA-Seq data with 50 million reads/sample. 23,043 genes (with average expression < 0.1 TPM) are divided into five ascending quintiles of expression based on their average expression in 155 samples. **(S9B)** Gene expression accuracy using data simulated with 1 million reads/sample, as a function of relative gene expression observed in actual RNA-Seq data with 50 million reads/sample. 23,043 genes (with average expression < 0.1 TPM) are divided into five ascending quintiles of expression based on their average expression in 155 samples.

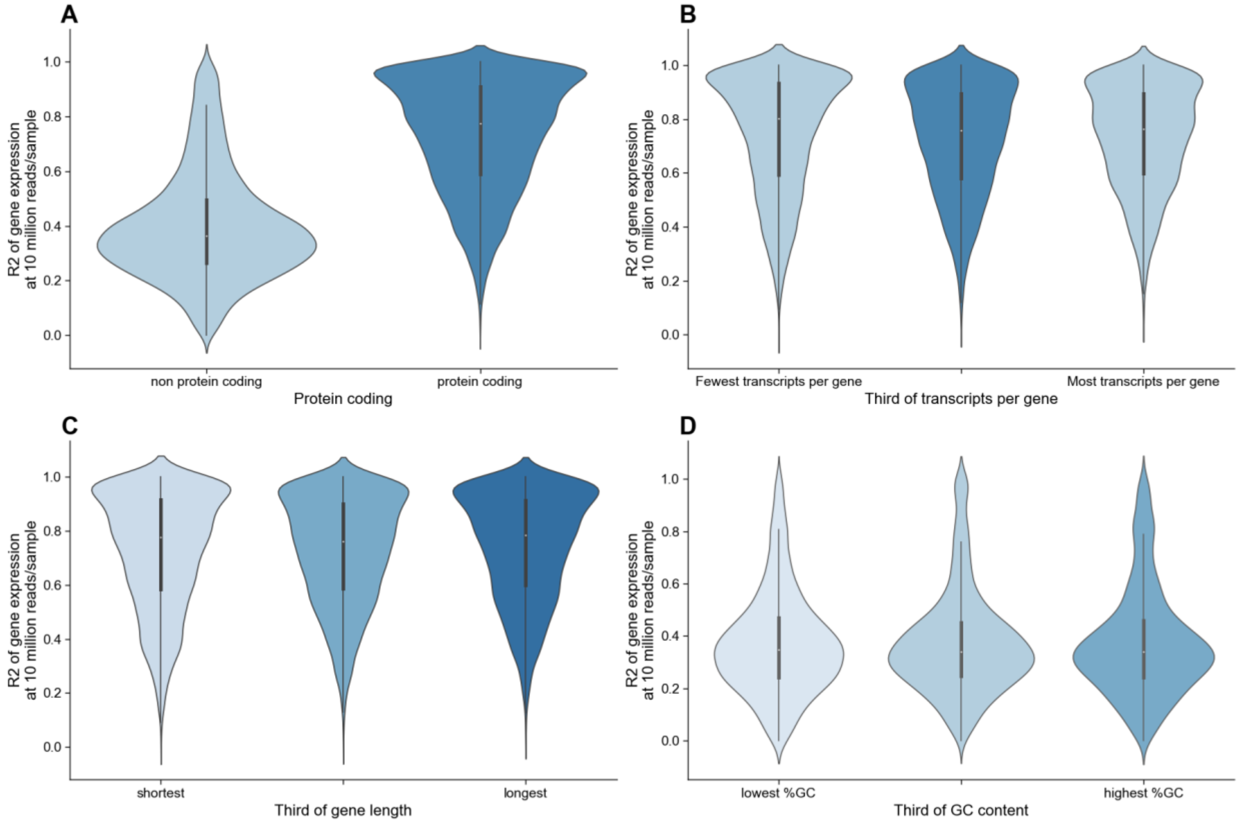

Figure S10: *Variability of correlation as a function of various gene characteristics*. **(S10A)** Gene expression estimation accuracy simulated at 10 million reads/sample as a function of whether a gene codes for a protein. 24,093 genes (with average expression < 0.1 TPM) are divided into two groups. **(S10B)** Gene expression estimation accuracy simulated at 10 million reads/sample as a function of how many transcripts each gene has. 23,540 genes (with average expression < 0.1 TPM) are divided into three ascending groups based on the number of transcripts contained in each gene. **(S10C)** Gene expression estimation accuracy simulated at 10 million reads/sample as a function of relative gene length. 14,484 genes (with average expression < 0.1 TPM, protein coding) are divided into three groups based on the length of each gene. **(S10D)** Gene expression accuracy as a function of relative GC content. 5,771 genes (with average expression < 0.1 TPM and GC content reported) are divided into three groups based on the length of each gene.

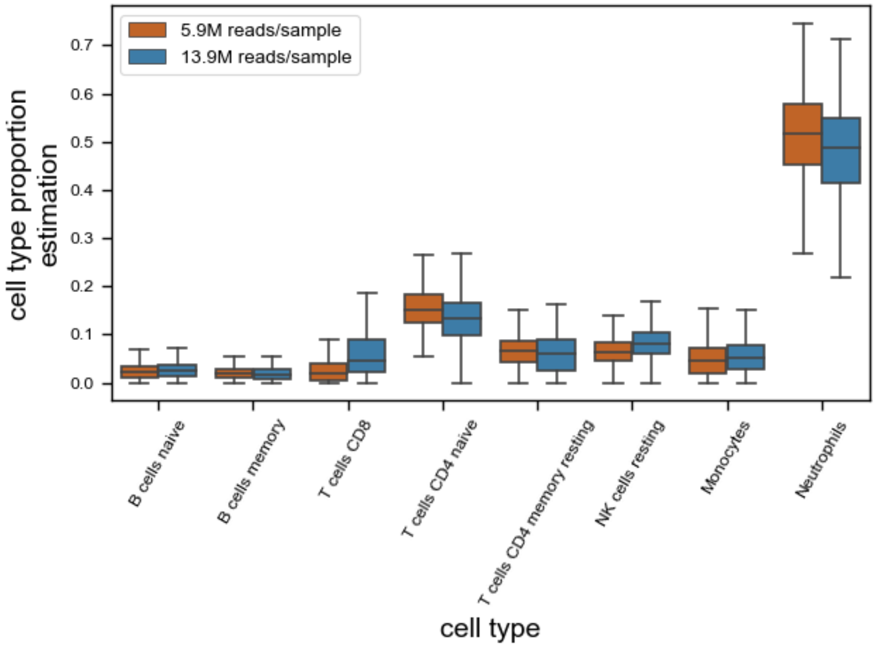

Figure S11: *Estimation of cell-type proportions.* **(S11)** In real data, a comparison of estimated cell type proportions from Cibersortx between lower-coverage (5.9M reads/sample) and moderate-coverage (13.9M reads/sample) RNA-Seq data for the eight most common cell types in whole blood tissue.

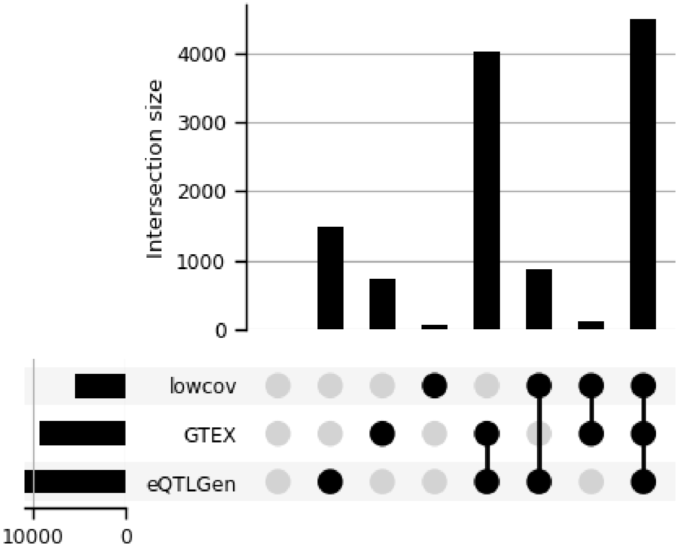

Figure S12: *Overlap of significant eGenes using RNA-Seq from three different datasets*. **(S12)** Comparing number of genes with significant associations between three datasets: (1) Lower-coverage RNA-Seq (5.9M reads/sample on average, across 1,496 individuals), (2) GTEX (83M reads/sample on average, across 670 samples), (3) eQTLGen (31,684 individuals, mix of RNA-Seq and MicroArray assays used).

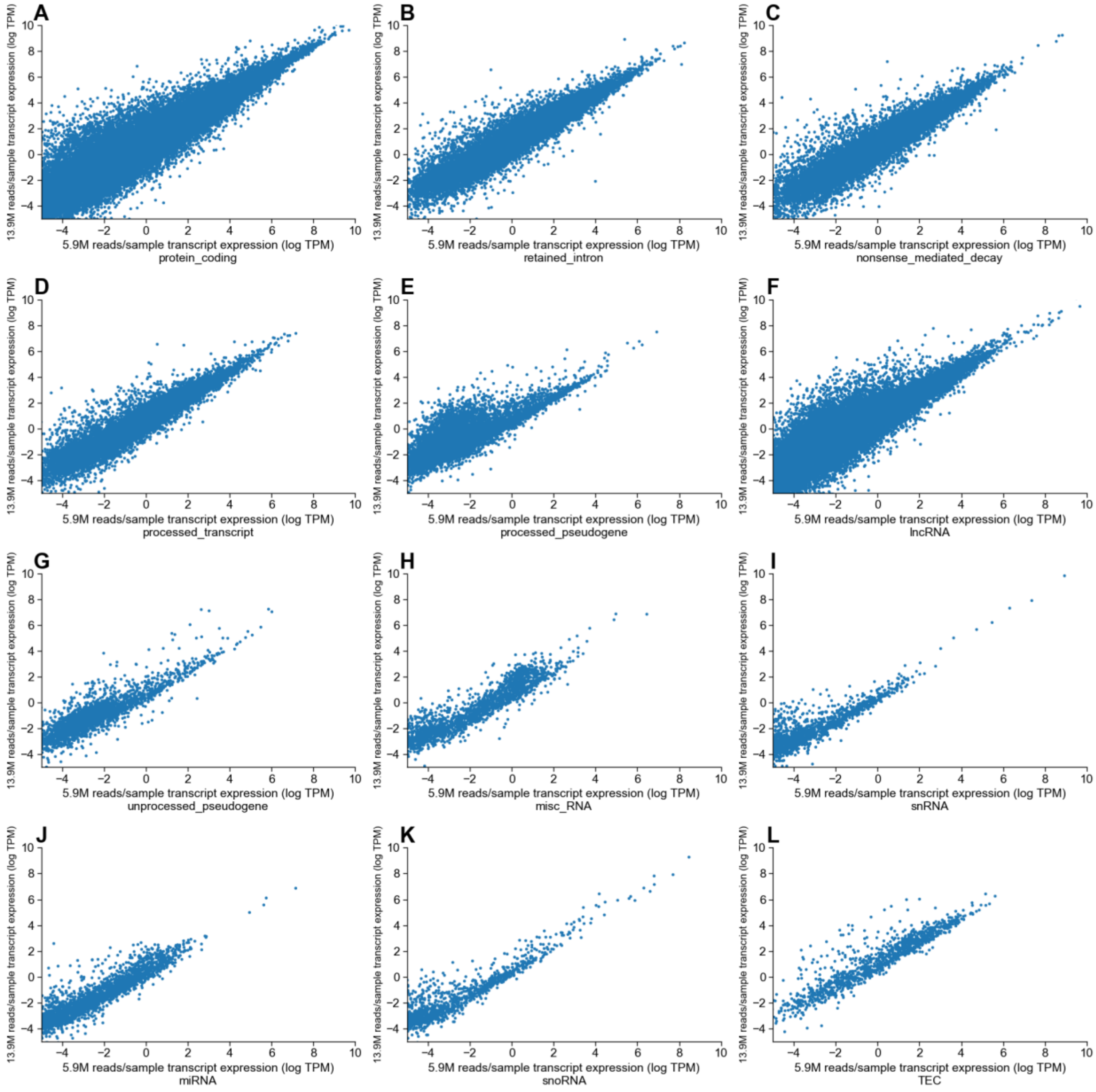

Figure S13: *Concordance of transcript expression estimation between lower-coverage RNA-Seq vs moderate-coverage RNA-Seq.* **(S13)** For the 12 most highly represented transcript types (> 1000 transcripts quantified), we show mean expression estimates in lower-coverage RNA-Seq (x-axis) versus moderate-coverage RNA-Seq (y-axis).

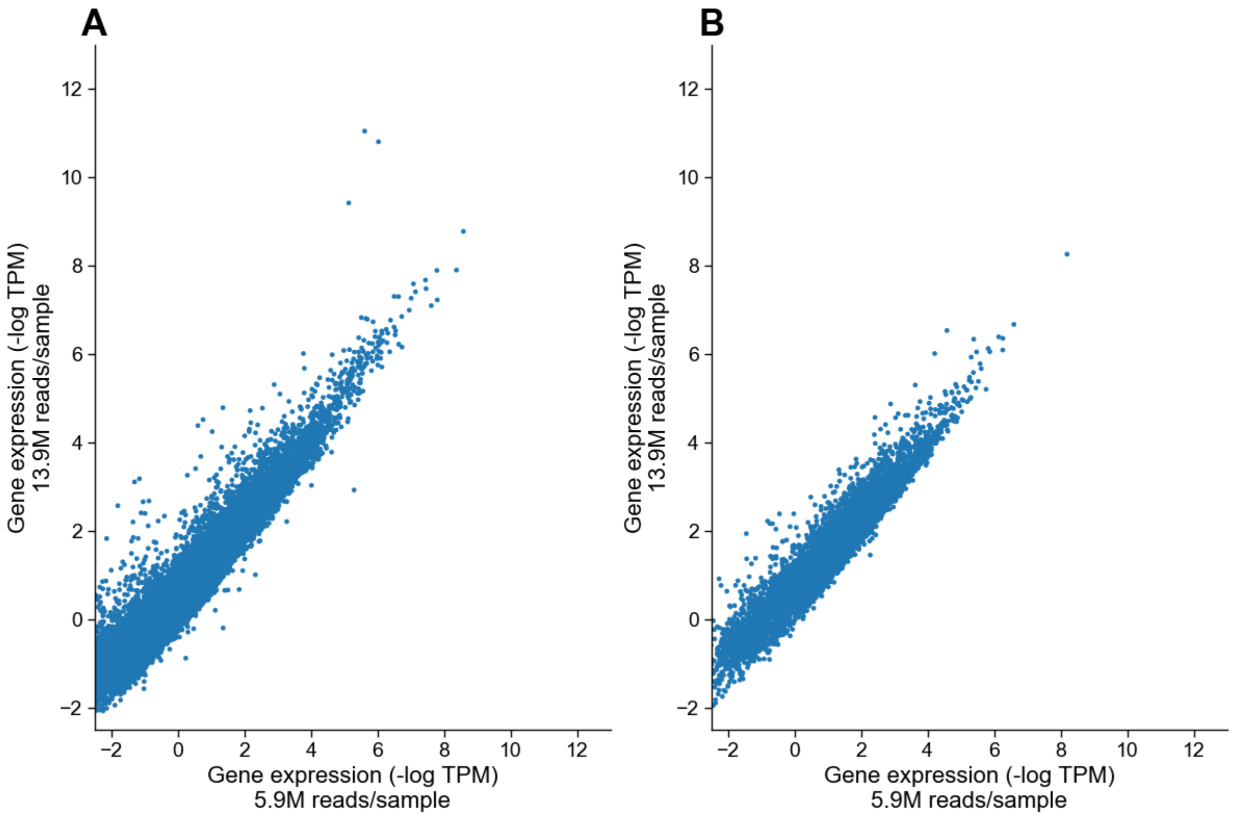

Figure S14: *Concordance of gene expression as a function of presence of a predominantly expressed transcript* **(S14A)** Restricting to the 5894 genes with a transcript responsible for at least 50% of the gene's expression in the 13.9M read/sample dataset, comparison of the mean expression (log TPM) across samples, of every gene. R^2 = 0.91. **(S14B)** Restricting to the 14711 genes without a transcript responsible for at least 50% of the gene's expression in the 13.9M read/sample dataset, comparison of the mean expression (log TPM) across samples, of every gene. R^2 = 0.90.

SUPPLEMENTARY TABLES

Platform	N Typed SNPs	N Imputed SNPs
OmniExpress Exome	619,690	5,576,428
Global Screening Array (GSA)	514,169	4,383,620
COEX	323,599	4,736,265
PsychArray	378,710	4,528,960
Merged	148,612	2,289,732

Table S1: *Number of typed and imputed SNPs after QC*

Dataset	Estimated number of total reads mapped in experiment	Estimated cost of experiment	Proportion of cost compared to lower-coverage RNA-Seq experiment	Proportion of eGenes identified compared to lower-coverage RNA-Seq
Lower-Coverage or M=5.9M reads/sample (Whole Blood)	~8.8B	~$292,000	1.0	1.0
GTEx	~55.6B	~$620,000	2.12	1.39

Table S2: *Whole blood RNA-Seq datasets and respective cost estimates*. We describe the lower-coverage and GTEx datasets in terms of estimated cost from our budget model and an estimate for total number of reads used. We assume that the cost of genotyping is $53 per sample (per UCLA Neurogenetics Sequencing Core).

Transcript type	Number of transcripts	Correlation (R^2) of expression
Protein coding	83735	0.92
Retained intron	28411	0.89
Nonsense mediate decay	15856	0.88
Processed transcript	14128	0.89
Processed pseudogene	10055	0.72
lncRNA	59133	0.85
Unprocessed pseudogene	2644	0.81
miscRNA	1987	0.82
snRNA	1730	0.95
miRNA	2757	0.81
snoRNA	1367	0.94
TEC	1135	0.82

Table S3: *Transcript-level expression correlations by transcript type.* We quantify expression for 226,390 transcripts. For the 12 transcript types with at least 1000 transcripts represented, we

calculate the correlations between mean transcript expression estimated using lower-coverage RNA-Seq and moderate-coverage RNA-Seq.

SUPPLEMENTARY NOTE

Notes about overlap in datasets:

- The samples in the low-coverage whole blood and high-coverage whole blood datasets are completely disjoint – no individuals overlap here.
- 97 individuals have RNA-seq data in both the high-coverage fibroblast dataset and low-coverage whole blood dataset
- 41 individuals have data in the high-coverage fibroblast dataset and the high-coverage whole blood dataset
- In total, 138 individuals overlap between the high-coverage fibroblast RNA-Seq samples and whole blood RNA-Seq samples (low-coverage and high-coverage)
