# Supplementary material for: Powerful eQTL mapping through low-coverage RNA sequencing
Source: HGG Adv. 2022 Apr 2;3(3):100103. doi: 10.1016/j.xhgg.2022.100103 (PMC9062329; doi:10.1016/j.xhgg.2022.100103)
Supplement: Document S2. Article plus supplemental information [file mmc2.pdf]

HGG Advances

ARTICLE

# Powerful eQTL mapping through low-coverage RNA sequencing

Tommer Schwarz,[1,2,*] Toni Boltz,[3] Kangcheng Hou,[1] Merel Bot,[4] Chenda Duan,[5] Loes Olde Loohuis,[7,8] Marco P. Boks,[9] René S. Kahn,[9,10] Roel A. Ophoff,[1,3,11] and Bogdan Pasaniuc[1,2,3,6,*]

## Summary

Mapping genetic variants that regulate gene expression (eQTL mapping) in large-scale RNA sequencing (RNA-seq) studies is often employed to understand functional consequences of regulatory variants. However, the high cost of RNA-seq limits sample size, sequencing depth, and, therefore, discovery power in eQTL studies. In this work, we demonstrate that, given a fixed budget, eQTL discovery power can be increased by lowering the sequencing depth per sample and increasing the number of individuals sequenced in the assay. We perform RNA-seq of whole-blood tissue across 1,490 individuals at low coverage (5.9 million reads/sample) and show that the effective power is higher than that of an RNA-seq study of 570 individuals at moderate coverage (13.9 million reads/sample). Next, we leverage synthetic datasets derived from real RNA-seq data (50 million reads/sample) to explore the interplay of coverage and number individuals in eQTL studies, and show that a 10-fold reduction in coverage leads to only a 2.5-fold reduction in statistical power to identify eQTLs. Our work suggests that lowering coverage while increasing the number of individuals in RNA-seq is an effective approach to increase discovery power in eQTL studies.

## Introduction

The vast majority of risk loci identified in genome-wide association studies (GWAS) are difficult to interpret as they lie in noncoding regions of the genome. Variants that regulate gene expression abundance, as measured through expression quantitative trait locus (eQTL) studies, provide insightful information about the functional interpretation of GWAS signals.[1,2] By integrating eQTL associations with GWAS, we can hope to identify target genes that are driving the GWAS signal at a locus.[3–6] RNA sequencing (RNA-seq) is the state-of-the-art assay for measuring gene expression in bulk tissue and is therefore the assay of choice for eQTL mapping.[7,8] However, the high cost of RNA-seq often limits the sample size and therefore reduces the discovery power of eQTL studies based on RNA-seq.[2,6,9] Recent work from the eQTLGen consortium, where they conducted a meta-*cis*-eQTL-analysis from 31,684 gene expression samples (combination of microarray and RNA-seq) and identified 16,987 eGenes. Consequent power analysis revealed that at a power of 0.80, 1,685 samples are needed to capture eGenes at an effect size of 0.124 (the median effect size observed among the 16,987 eGenes identified in the study).[10]

Traditional RNA-seq study design prioritizes sequencing depth per individual (targeted levels of coverage in the range of 30–50 million reads) over the number of individuals (samples) included in the study.[11–14] However, given that high levels of coverage per individual limits the sample size of a study, this results in a loss of statistical power in eQTL mapping. Previous studies have established that the low-coverage whole-genome sequencing of a larger number of individuals attains increased power of association compared with higher-coverage studies of smaller sample sizes in GWAS.[15–19] This raises the hypothesis that, similarly as for whole-genome sequencing and GWAS, lower-coverage RNA-seq with a considerable increase in the number of individuals sequenced could increase power of discovery in eQTL studies.[20–24] Currently, there is no systematic approach for determining the optimal sample size (in terms of number of sequenced individuals) and coverage to maximize eQTL discovery power.

One application of eQTL discovery is integration with GWAS, using methods such as coloc,[25] to better understand biological mechanisms driving these GWAS loci. Recent work from GTEx shows that just ∼20% of GWAS loci colocalize with eQTLs in the most relevant tissue to the trait, and other work shows that an average of just ∼11% of trait narrow-sense heritability is explained by *cis*-eQTLs measured in GTEx.[26–28] To better characterize GWAS loci, it is clear that large sample sizes are especially necessary for maximizing power in eQTL studies.[10]

[1]Bioinformatics Interdepartmental Program, University of California Los Angeles, Los Angeles, CA, USA; [2]Department of Computational Medicine, David Geffen School of Medicine, University of California Los Angeles, Los Angeles, CA, USA; [3]Department of Human Genetics, David Geffen School of Medicine, University of California Los Angeles, Los Angeles, CA, USA; [4]Center for Neurobehavioral Genetics, Semel Institute for Neuroscience and Human Behavior, University of California, Los Angeles, CA, USA; [5]Department of Computer Science, University of California, Los Angeles, Los Angeles, CA, USA; [6]Department of Pathology and Laboratory Medicine, David Geffen School of Medicine, University of California Los Angeles, Los Angeles, CA, USA; [7]Department of Psychiatry, David Geffen School of Medicine, University of California, Los Angeles, Los Angeles, CA, USA; [8]Program in Neurobehavioral Genetics, Semel Institute, David Geffen School of Medicine, University of California, Los Angeles, Los Angeles, CA, USA; [9]Department of Psychiatry, Brain Center University Medical Center Utrecht, University Utrecht, Utrecht, the Netherlands; [10]Department of Psychiatry, Icahn School of Medicine, Mount Sinai, NY, USA; [11]Department of Psychiatry, Erasmus University Medical Center, Rotterdam, the Netherlands

*Correspondence: tomschwarz@g.ucla.edu (T.S.), pasaniuc@ucla.edu (B.P.)
https://doi.org/10.1016/j.xhgg.2022.100103.
© 2022 The Authors. This is an open access article under the CC BY license (http://creativecommons.org/licenses/by/4.0/).

**Table 1. RNA-seq datasets discussed in this paper**

| Referred to as: | Coverage (million reads/sample) | Tissue | No. of samples | Library prep method |
|---|---|---|---|---|
| Lower-coverage or M = 5.9 million reads/sample (whole blood) | 5.9 | whole blood | 1,490 | TruSeq Stranded plus rRNA and GlobinZero |
| Moderate-coverage or M = 13.9 million reads/sample (whole blood)[19] | 13.9 | whole blood | 570 | meta-analysis of (1) TruSeq Stranded plus rRNA and GlobinZero and (2) TruSeq Stranded polyA selected |
| High-coverage (fibroblast) | 50.3 | fibroblast | 150 | TruSeq Stranded polyA selected |
| GTEx[12] | 82 | whole blood | 670 | TruSeq Non-stranded polyA selected |
| eQTLGen[13] | N/A | whole blood | 31,684 | meta-analysis consisting of RNA-seq and microarray |

The coverage refers to the average number of reads that successfully map to the transcriptome, except for GTEX, which refers to the median number of total reads/sample (average mapped not available). Further description of sample overlaps among cohorts in supplemental note.

Looking back over the past decade since the inception of RNA-seq, the size of RNA-seq datasets has been steadily increasing as a result of decreasing sequencing costs and an emphasis on exploring the biological mechanisms behind GWAS hits.[29] Moving forward, as this trend continues, RNA-seq experiment design is a critical part of maximizing data resources.[30]

In this work, we perform RNA-seq in 1,490 individuals at a lower coverage (average mapped read depth of 5.9 million reads/sample) and find that eQTL discovery power is better than that of an experiment with a similar budget, but with fewer individuals and higher coverage. Compared with moderate-coverage RNA-seq[31] and GTEx, we find a high degree of consistency in both the gene expression as well as eQTL effects. We assess the interplay of coverage per sample and accuracy of expression estimates using synthetic RNA-seq datasets generated by the downsampling of real high-coverage data (50 million reads/sample). In addition, we generate synthetic data derived from an RNA-seq experiment done at 50 million reads/sampleuse these synthetic datasets to precisely show how decreasing coverage affects accuracy of gene quantification overall, and in different gene categories (by expression, numbers of transcripts, gene length, etc.). Our analyses show that a sequencing experiment conducted with a target coverage of 10 million reads/sample has an average correlation per gene of 0.40 when compared with an experiment conducted with a target coverage of 50 million reads/sample. We provide evidence to show that, under a fixed budget, sequencing at lower-coverage levels (<10 million reads/sample) and increased sample size can boost the effective sample size per unit of cost compared with standard approaches of eQTL study design.

## Results

### Low-coverage RNA-seq is accurate for eQTL mapping

To validate the utility of low-coverage RNA-seq, we sequenced whole-blood tissue from N = 1,490 unrelated individuals (materials and methods) (Figures S1A and S1B). We target a sequencing coverage of 9.5 million reads/sample, yielding M = 5.9 million reads mapped to RefSeq genes, on average (SD across samples of 1.96 million, Figure S2). We refer to this dataset as the lower-coverage RNA-seq, or the M = 5.9 million reads/sample dataset. We contrast this dataset with an RNA-seq dataset obtained with a similar budget, but with 2.4-fold higher coverage (M = 13.9 reads) across N = 570 individuals (Figures S1C and S1D).[22] We refer to this as the moderate-coverage whole-blood RNA-seq, or the M = 13.9 million reads/sample dataset (Table 1).

First, we assess the number of genes quantified in the two datasets. We observe 40,459 genes with at least one mapped read on average across samples in the whole-blood moderate-coverage dataset, and 27,308 genes with at least one mapped read on average across samples in the whole-blood lower-coverage dataset. Notably, when restricting to protein coding genes with at least one mapped read in both the moderate-coverage and lower-coverage datasets, we find more similar numbers between the datasets, with 18,329 and 15,605 genes quantified, respectively. This is likely due to the very sparse abundance of the non-protein coding genes, making them less likely to be detected in a lower-coverage dataset. Indeed, we observe similar effects across the moderate- versus low-coverage datasets when assessing the genes with sufficient expression to be included in eQTL analysis (TPM > 0.1 in 20% of individuals, see materials and methods): 26,566 genes (15,496 protein coding genes) in moderate-coverage data versus 19,039 (13,339 protein coding genes) in lower-coverage data. Most importantly, we observe a high correlation in the abundance levels across the two datasets. We calculate the median TPM across samples of 62,487 gencode genes and restrict to the 20,735 protein coding genes that are detected in both datasets. Without recalculating TPM after these restrictions we observe a Pearson correlation ($R^2$) of 0.91, thus demonstrating that moderate- and lower-coverage RNA-seq recover similar expression (Figure 1A).

Next, we investigate the power of low-coverage RNA-seq for eQTL mapping. We conducted *cis*-eQTL mapping with a 1 Mb window using FastQTL, restricting to the 1,490

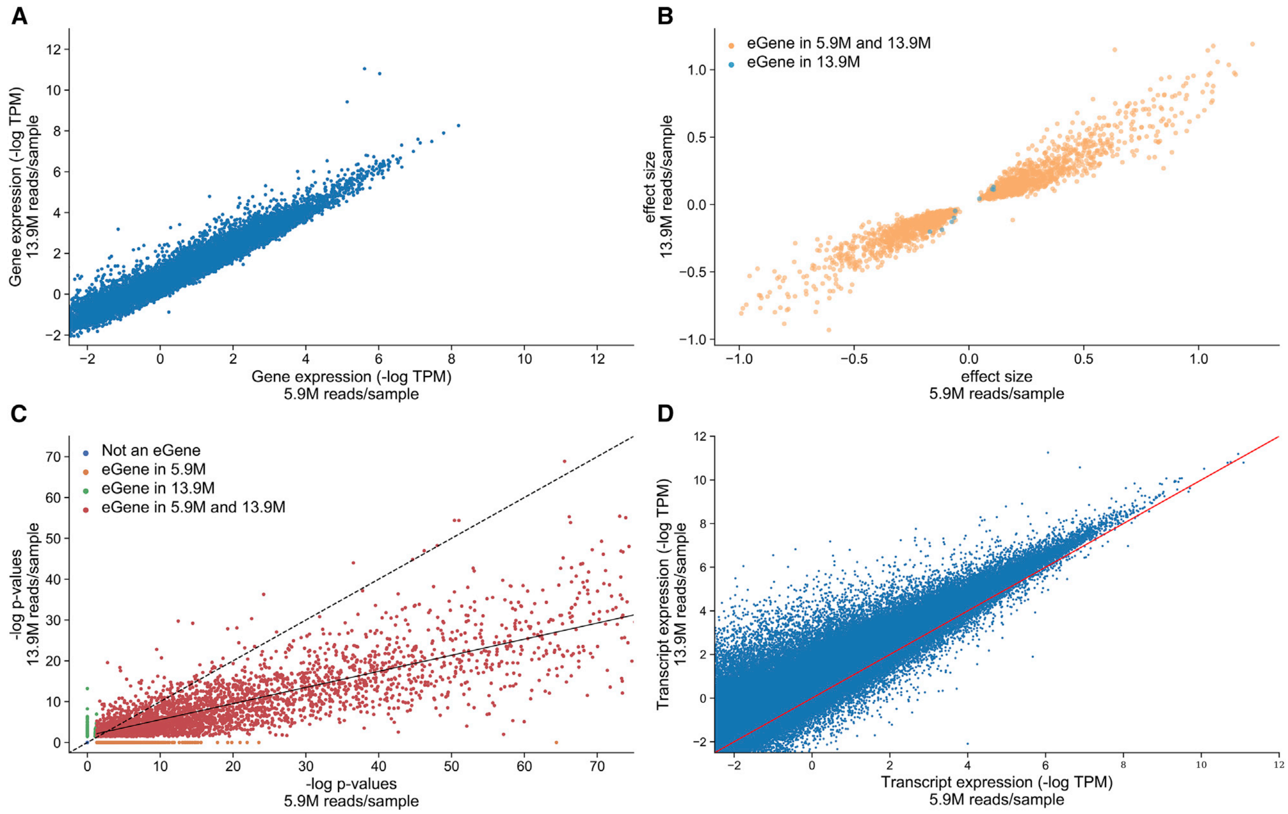


**Figure 1. Concordance of eQTL discovery when using lower-coverage RNA-seq versus moderate-coverage RNA-seq**
(A) Restricting to the 20,735 genes with sufficient expression levels to be included in eQTL analysis in both the 5.9 and 13.9 million reads/sample dataset, comparison of the median expression (log TPM) across samples, of every gene. $R^2 = 0.91$.
(B) In real data, scatterplot of effect sizes of most significant eQTL hits for the 2,151 protein coding genes with the same eQTL hit in both eQTL analyses performed (lower coverage and moderate coverage). On the x axis, we show the effect sizes for these genes using lower-coverage RNA-seq, on the y axis we show the effect sizes for these genes using moderate-coverage RNA-seq.
(C) Real data p value comparison scatterplot: in real data, scatterplot of –log p values of most significant eQTL hit for 13,950 genes included in both eQTL analyses performed (lower coverage and moderate coverage). On the x axis, we show the –log p values for these genes using lower-coverage RNA-seq, on the y axis we show the –log p values for these genes using moderate-coverage RNA-seq. The dotted line shows $y = x$, while the solid line shows the line of best fit for the 3,985 protein coding eGenes with a significant eQTL hit in both datasets.
(D) For the 227,046 unique isoforms detected in the lower-coverage and moderate-coverage datasets, we show the mean expression across samples in each dataset ($R^2 = 0.83$).

unrelated individuals in the lower-coverage RNA-seq data (materials and methods), to identify 7,587 genes (eGenes) with a significant association at an FDR adjusted p-value $< 0.05$ . As expected, eQTL distribution is concentrated at TSSs, with 73% of eGenes TSS within 250 kb of the associated SNP (eSNP). Repeating this approach using the moderate-coverage whole-blood data in 570 individuals, we only find 5,971 genes with a significant association at an FDR adjusted p-value $< 0.05$ . A total of 4,969 of the 7,587 eGenes found using the lower-coverage data are also significant in the moderate-coverage data. Of these, 2,163 of the eGenes are protein coding eGenes that share the same associated eSNP, and we see an extremely high level of concordance between effect sizes for these eGenes across the two datasets ($R^2 = 0.93$, Figure 1B). This further indicates that low-coverage RNA-seq is robust in capturing eQTL effect sizes. In brief, we tested to see whether the mean expression or number of transcripts differed between eGenes that shared the same eSNP between the two datasets ($n = 2{,}163$) and those that did not ($n = 4{,}324$) (Figure S3). We find slightly higher expression and a slight increase in the number of transcripts in the set of eGenes that do share the same eSNP. A total of 1,002 genes were found to be eGenes in the moderate-coverage eQTL analysis but not in the lower-coverage analysis, with 573 (of the 1,002) not passing expression levels (TPM $> 0.1$ in 20% individuals) to be included in the lower-coverage eQTL analysis; only 234 of the 573 were protein coding genes, suggesting that, for most protein coding genes, lower-coverage RNA-seq can adequately capture their expression. Similar concordance is observed between p values for the associations in both datasets (Figure 1C). Comparing the p values for eGenes detected in both eQTL analyses, the corresponding regression line has a slope of 0.39, consistent with the lower-coverage dataset having superior statistical power to detect associations over the moderate-coverage dataset, and consistent with overall number of significant eQTL discoveries. We report

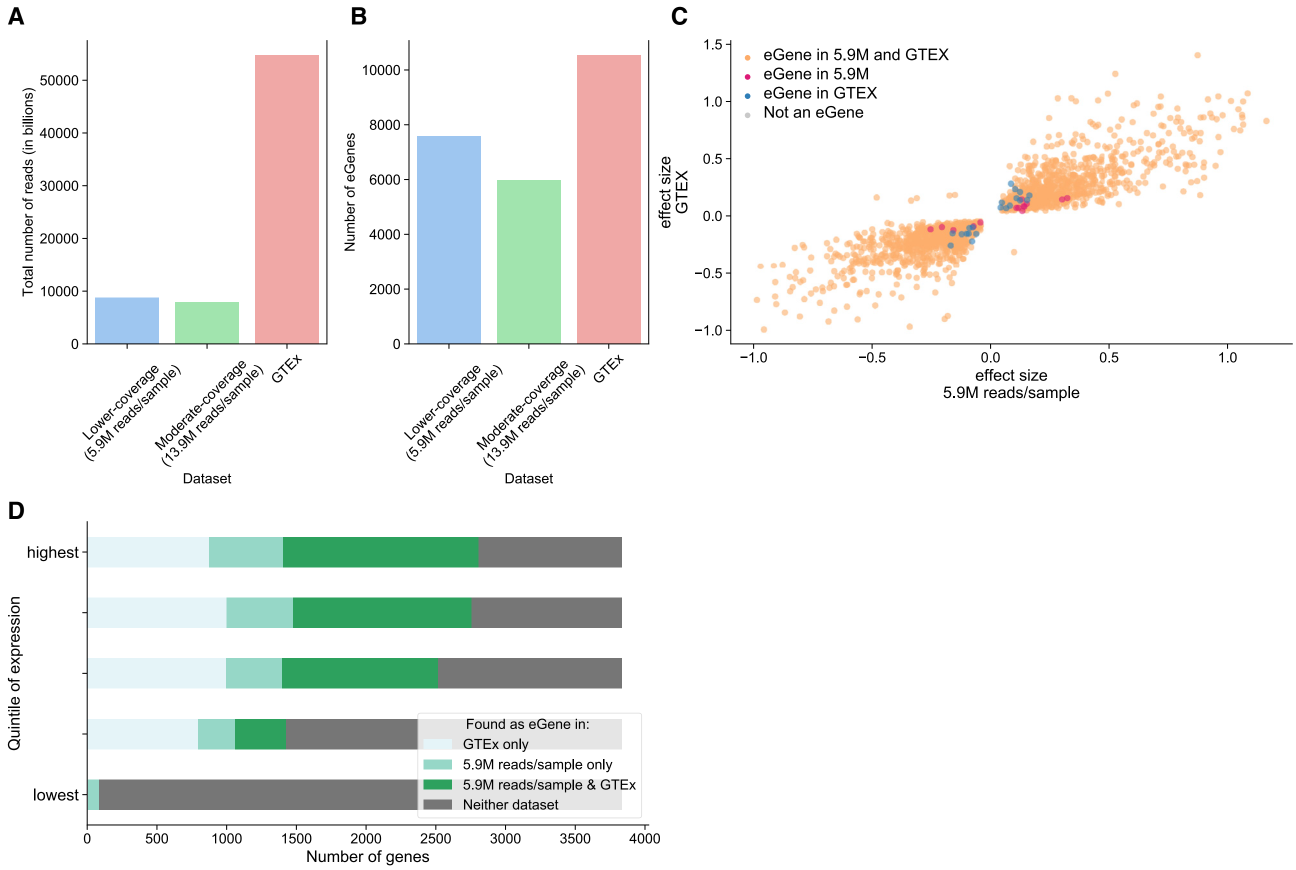


**Figure 2. eQTL analysis using lower-coverage RNA-seq is comparable with eQTL analysis from the GTEX Consortium**
(A) Estimates for the total number of reads (in billions) included in each of the three RNA-seq experiments that we compare.
(B) Number of eGenes discovered at an FDR correction level of 0.05 in each of the three datasets that we compare.
(C) In real data, scatterplot of effect sizes of the most significant eQTL hit for the 1,927 eGenes with leading eSNPs in LD with $R^2 > 0.25$ between the two datasets (lower-coverage RNA-seq with 5.9 million reads/sample and GTEX). On the x axis, we show the effect size for these eGenes from eQTL analysis conducted using the 1,490 individuals of EUR ancestry and typed genotypes, and on the y axis we show the effect sizes for these eGenes from eQTL analysis published by the GTEX Consortium.
(D) The overlap in eGenes identified in the lower-coverage RNA-seq and GTEX, stratified into quintiles by the mean expression level observed in GTEX.

the results from using typed SNPs in these eQTL analyses (materials and methods), but observe similar patterns when using the full set of imputed SNPs.

More recently, RNA-seq data has been used to quantify gene expression at different resolutions, specifically at the transcript/isoform levels. To investigate whether lower-coverage RNA-seq can be reliably used in this context, we use kallisto[33] to quantify transcript expression in both the 5.9 and 13.9 million reads/sample datasets (materials and methods). We quantify 227,046 transcripts between the two datasets and find strong concordance between transcript expression estimates across them ($R^2 = 0.83$), suggesting that lowering coverage to this degree does not strongly influence the ability to detect changes in transcript expression (Figure 1D). However, there does seem to be associations between transcript type and how well the transcript is quantified using lower-coverage RNA-seq (Figure S13; Table S3).

To further validate the performance of eQTL analysis using low-coverage RNA-seq (coverage 5.9 M, n = 1,490), we compared the resulting eQTLs to the ones found by GTEx in whole blood[13] (Figure 2). Restricting to the 12,247 protein coding genes with sufficient expression to be included in both studies (>0.1 TPM in 20% of samples) we find that 3,916 out of the 5,538 protein coding genes (71%) with a significant association using the lower-coverage data also had a significant association in GTEx, correcting at an FDR adjusted p-value $< 0.05$ . We note that this is not an entirely equal comparison as the three datasets are generated from different budgets (Table S2). While GTEX (n = 668, 82 million reads/sample) consists of 55.6 billion reads, the lower-coverage (n = 1,490, 5.9 million reads/sample) and moderate-coverage (n = 570, 13.9 million reads/sample) datasets consist of just 8.8 and 7.9 billion reads, respectively. Considering the number of eGenes discovered using each of these datasets, we find that per 1 billion total reads, we discover 862 eGenes using the lower-coverage dataset, 756 eGenes using the moderate-coverage dataset, and just 190 eGenes in GTEx (Figures 2A and 2B). Among eGenes shared by both

**Table 2. Sequencing cost scenarios**

| | Cost per lane ($) | Cost per sample ($) |
|---|---|---|
| Scenario 1 | 1,790 | 87 |
| Scenario 2 | 1,790 | 30 |
| Scenario 3 | 1,790 | 150 |
| Scenario 4 | 1,000 | 150 |

The cost parameters corresponding to the effective sample size scenarios in Figure 4. Cost per sample reflects the cost of library prep to include an additional sample. Cost per lane reflects the cost per sequencing lane, which allows for 300 million reads.

datasets, we found that the leading eSNPs are in LD (average $R^2 = 0.41$, $SD = 0.39$), showing that lower-coverage RNA-seq captures the same eQTL signal, either directly or by a nearby tagged SNP. Further restricting to eGenes with leading eSNPs with an LD $R^2$ value of at least 0.25 in both of these datasets (1,927 genes) (Figure 2C), we observe a correlation ($R^2$) of 0.81 between their effect sizes. We find consistently high correlations regardless of the LD threshold used here (Figure S4). Looking into the 1,622 protein coding genes with a significant association in eQTL analysis using the lower-coverage RNA-seq but not in GTEx using an FDR adjusted p-value cutoff of 0.05, we observe that 283 have a significant association in GTEx using an an FDR adjusted p-value cutoff of 0.10 . To further ensure that these eGenes are not false positives, we compare the set of 1,622 genes with eQTL analysis conducted by the eQTLGen Consortium[10] and find that 1,498 of these genes (92.4%) have been found to have a significant association in eQTLGen. This suggests that the additional associations found using lower-coverage data that are not found in GTEx are not false positives, but fall just below the significance threshold in the GTEx analysis.

Next, we investigate whether lower-coverage RNA-seq "misses" genes with a low overall expression due to sequencing bias. To do this, we stratify the 19,175 protein coding genes measured in GTEX into five groups by mean expression and report how many genes from each of these groups are discovered as eGenes using (1) GTEx, (2) lower-coverage sequencing, (3) both datasets, and (4) neither dataset (Figure 2D). At the lowest quintile of expression (3,835 genes total), we observe that GTEx reports just 6 of these genes as eGenes, while using lower-coverage sequencing reports 78 to be eGenes. In the other four quintiles of higher expression, we observe fairly consistent numbers of eGenes identified only in GTEx (794, 876, 997, 1,000, in increasing order), indicating that the lower-coverage sequencing performs consistently across coveragegene abundance levels. We perform an analogous analysis comparing GTEx and the moderate-coverage dataset (Figure S6A), and find that the moderate-coverage RNA-seq also does not detect many eGenes from the lowest expressed quintile of genes.

Next, we look at whether the effect size comparison in real data between eGenes discovered using lower coverage and moderate coverage data is inflated due to poor estimation of lowly expressed genes in both datasets. Similarly to the previous section, we stratify the 19,175 protein coding genes measured in GTEx into five groups by mean expression and report how many genes from each of these groups are discovered as eGenes using (1) moderate-coverage, (2) lower-coverage RNA-seq, (3) both datasets, and (4) neither dataset (Figure S6B). If the effect size concordance was in fact inflated, in real data we would see either a lot of shared detected or shared missed eGenes among the lowly expressed gene quintiles in the lower- and moderate-coverage data that are detected in GTEx. However, Figure S6B shows that none of the three datasets reliably detect eQTLs in the quintile of lowest expression.

To demonstrate that these eQTLs are implicated in GWAS loci, we run colocalization analysis using GWAS statistics from several blood traits (mean corpuscular volume, mean cell hemoglobin, and systemic lupus) (Table 2). Using a PP4 threshold of 0.80 (materials and methods), we see that a total of 51 unique eGenes (0.67% of significant associations) colocalize with a total of 50 unique GWAS SNPs. This is especially encouraging, as we see that there does not exist a redundancy of GWAS loci explained by eQTL hits. When performing the same analysis using data from GTEx, we find that a total of 91 unique eGenes (0.86% of significant associations) colocalize with 82 unique GWAS SNPs. Fourteen eGenes are in common with five GWAS SNPs involved in a significant colocalization in both datasets.

We perform a TWAS analysis for the same three traits (Table 3) and find that, using the lower-coverage data, there are 143 significant TWAS associations. Using GTEx, there are 311 significant TWAS associations. Between the two datasets, 59 eGenes are shared.

Finally, we explore the impact of RNA-seq at lower coverages for cell-type expression estimation. We use CIBERSORTx[44] to compare cell-type proportion estimates between the lower-coverage data and moderate-coverage data (materials and methods). We find that the median estimated cell-type proportions are conserved across both datasets, suggesting that deconvolution of cell-type-specific signals from gene expression profiles of whole-blood samples is not impacted when coverage is reduced by half (Figure S11).

### Impact of RNA-seq coverage on eQTL power

Having demonstrated the accuracy of low-coverage RNA-seq in eQTL mapping in real data, we next focused on exploring the interplay of number of individuals and coverage for optimizing power for discovery in this eQTL study. As simulating RNA-seq data is challenging,[34,35] we downsample reads from high-coverage RNA-seq data to create synthetic datasets at various coverages (materials and methods). We observe that, with just a fraction of the reads, it is still possible to estimate gene expression (Figure 3A). For example, we demonstrate, using synthetic data, that using just 10% of the data (5.0 million reads/sample) retains a per gene $R^2$ of 0.40, on average. In

**Table 3. Coloc results for selected blood traits**

| Trait | n coloc eGenes – lower-coverage (PP4 > 0.8) | n coloc GWAS SNPs – lower-coverage (PP4 > 0.8) | n coloc eGenes – GTEx (PP4 > 0.8) | n coloc GWAS SNPs – GTEx (PP4 > 0.8) |
|---|---|---|---|---|
| Mean corpuscular volume | 36 | 27 | 54 | 45 |
| Mean cell hemoglobin | 33 | 29 | 52 | 42 |
| Systemic lupus | 6 | 6 | 22 | 11 |
| All of the above | 51 | 50 | 91 | 82 |

The number of unique eGenes (columns 1 and 3) and GWAS SNPs (columns 2 and 4) with PP4 > 0.80 when running colocalization analysis on significant eQTLs from analyses using lower-coverage RNA-seq (columns 1 and 2) and results from GTEx (columns 3 and 4).

practice, increasing the number of samples in an RNA-seq study leads to increased library preparation costs, making the increase in obtainable statistical association power less obvious.

It has been established that statistical power in association studies is a function of sample size, phenotype measurement accuracy, and genotype measurement accuracy.[15,16,21,35] This means that the power of a study with sample size N and estimated gene expression is approximately the same as the power of a study with sample size $R^2$*N, using the true gene expression measurements (materials and methods). In this scenario, $R^2$ is the correlation between the true expression and the expression estimates. We therefore report the squared correlation ($R^2$) between synthetic datasets at various coverages and the full data at an average of 50 million reads/sample (which is assumed to be the true gene expression). While these results show the mean $R^2$ for all genes obtained under one synthetic dataset (one draw) per coverage level, we find that the synthetic datasets are consistent across multiple draws at the same coverage level (Figure S8A) and each show similar correlations with the ground truth gene expression (Figure S8B).

Next, we quantified how well lower-coverage RNA-seq can be used to detect eGenes. We explore the number of genes with significant associations after FDR correction at 5% under various levels of simulated coverage (Figure 3B). Using synthetic data, as the number of reads/sample decreases, we find that many eGenes are still detectable. For example, at 10 million reads/sample, just 20% of the full coverage, 60% of the eGenes are still detected. In the context of eQTL studies, synthetic RNA-seq supports the idea that sequencing at lower coverages over a higher number of individuals is a promising approach to boosting statistical power.

Finally, we explore the estimation accuracy in the synthetic data as a function of relative gene expression abundance, since less-abundant genes may not be captured altogether at lower sequencing coverages. We stratify genes into five groups based on their relative expression in the full dataset (M = 50.3 million reads/sample) and report the $R^2$ for genes in each of these groups in synthetic data (Figure 3C). We observe that, in the synthetic RNA-seq dataset at 10 million reads/sample, we capture expression of highly expressed genes better than lower-expressed genes. Specifically, for genes in the lowest through the highest quintiles of relative gene abundance, we find the average correlation ($R^2$) to the ground truth of expression to be 0.36, 0.44, 0.61, 0.73, and 0.86, respectively. We observe the same effect for synthetic datasets at coverages of 1 and 25 million reads/sample (Figures S9A and S9B). These results suggest that the ability to achieve similar power in eQTL analysis studies will differ per gene, and is a function of relative expression. We further investigate the properties of genes with quantification accuracy influenced by coverage levels of sequencing and find that that protein coding genes are more accurately quantified at lower-coverage levels compared with non-protein coding genes (Figure S10A). Conversely, the number of transcripts per gene, gene length, and GC content do not appear to be factors that broadly influence the gene quantification accuracy when sequencing coverage is reduced (Figures S10B, S10C, and S10D). We also investigate in real data whether genes with a predominantly expressed transcript are better estimated in lower-coverage data compared with those genes that do not have a predominantly expressed transcript (Figure S14). We do not find that this is a factor that strongly impacts gene quantification accuracy in real data.

### Optimal association power for eQTLs is attained at lower coverage with a larger number of samples

In the context of reducing experimental costs, we explored the trade-off between the number of samples sequenced and the average coverage per sample. To further evaluate the ability of lower-coverage sequencing to recapitulate expression signal observed in high-coverage data, we evaluated the expected effective sample size obtained with lower coverages per sample compared with a conventional approach of 50 million reads/sample. We downsample reads (materials and methods) from a high-coverage RNA-seq experiment derived from fibroblast tissue to create lower-coverage RNA-seq synthetic data. This is done to match actual low-coverage sequencing as closely as possible. To evaluate the relationship between cost, coverage, and sample size, we use the following equation to model the budget: $B = n*e+n*g+n*a+\frac{n*b*c}{d}+f$ (materials and methods).

We compute the effective sample size of an eQTL study as a function of average coverage, which determines the number of samples sequenced under a fixed budget

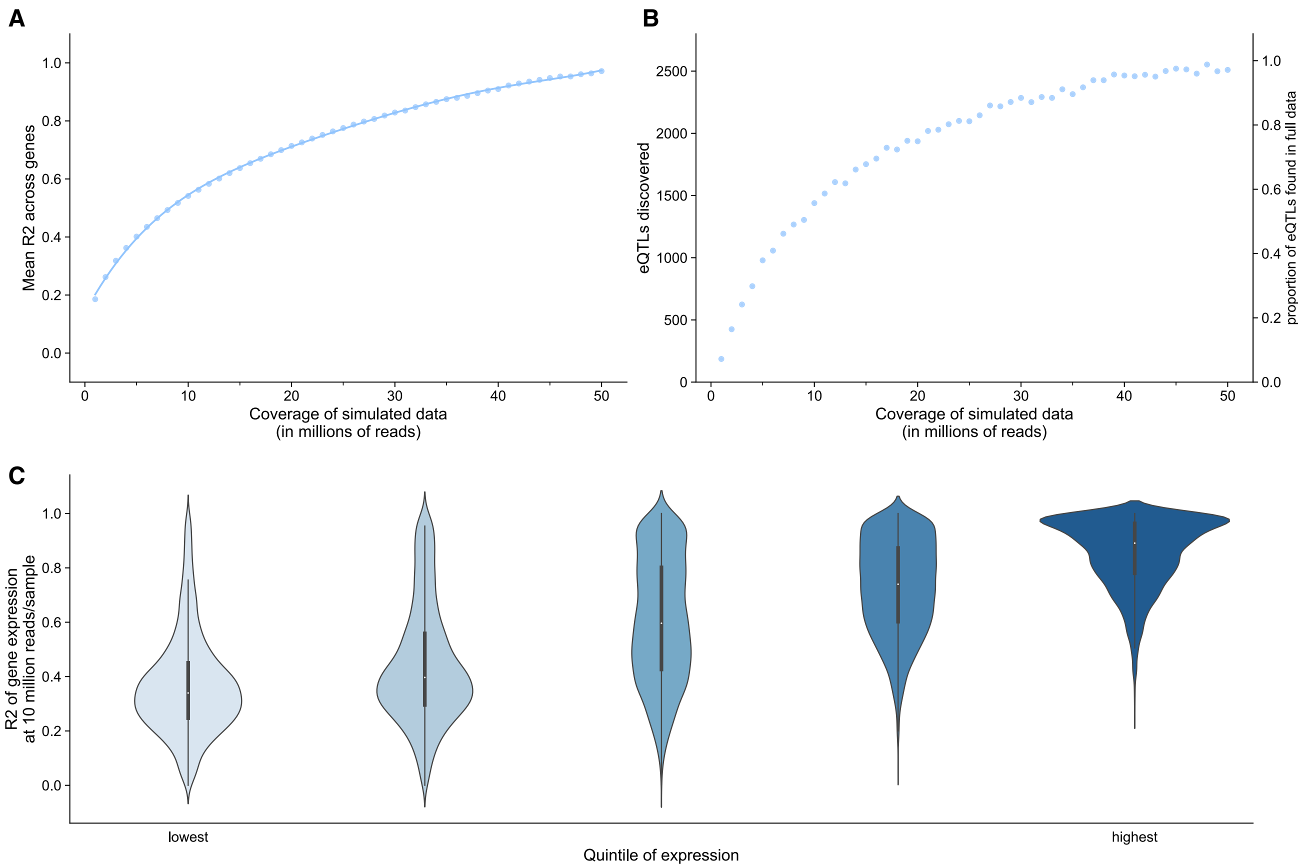


**Figure 3. Synthetic lower-coverage RNA-seq captures expression signal**
(A) On the x axis, we show the level of simulated coverage, and on the y axis we show the mean Pearson correlation of every gene. We calculate this value by finding the $R^2$ values for the TPM values of each of 45,910 genes across 155 samples between the high-coverage data (average of 50 million reads/sample) and the simulated data, and reporting the mean $R^2$ value per gene.
(B) For a fixed number of individuals, absolute number and percentage of eGenes captured at 5% FDR for synthetic RNA-seq at varying levels of coverage.
(C) Gene expression accuracy as a function of relative gene expression observed in actual RNA-seq data with 50 million reads/sample. 23,540 genes (with average expression >0.1 TPM) are divided into 5 ascending quintiles of expression based on their average expression in 155 samples.

(Figure 4A, corresponding cost parameters in Table 4). As an example, at a fixed budget of $300,000, the highest effective sample size is achieved by sequencing 1,378 individuals using 13 million reads/sample, which leads to a corresponding effective sample size of 877. An experiment achieving the sample effective sample size, using 50 million reads/sample, would cost $384,418 (N = 877, $R^2 = 1.0$). Therefore, by lowering the coverage of each sample and increasing sample size, we achieve the same effective sample size at just 78.0% of the cost. In practice, it is common to observe a considerable discrepancy between the target number of reads in an experiment and the number of reads that successfully map to genes. This can be attributed to different library prep techniques, quality of samples, or tissue type. To show how mapping rate can influence the effective sample size of an experiment, we model effective sample size with varying levels of mapping rates (materials and methods). As expected, we observe that, as the mapping rate increases, there is a corresponding increase in effective sample size (Figure 4C).

With a budget of ~$300,000 and an expected mapping rate of 0.60 (chosen based on the mapping rate of similar experiments using TruSeq Stranded plus rRNA and GlobinZero in whole-blood tissue), we see that the maximum effective sample size would be achieved at a target coverage of 16 million reads/sample, including 1,274 individuals in the study. We estimate that achieving the same effective sample size using data with 50 million reads/sample would cost ~$320,000 (N = 723), or 1.06× the cost of sequencing 1,274 individuals at a coverage of 16 million reads/sample. To explore other cost scenarios, we created a webtool where one can enter budget, costs, and other details about the experiment to see how to achieve optimal effective sample size (see web resources).

We use this budget model to calculate the cost of the eQTL analysis performed by GTEx under standard cost assumptions (materials and methods). We find that the cost of this experiment (n = 668, 82 million reads/sample on average) would have been ~$620,000. The cost of the lower-coverage RNA-seq (n = 1,490, 5.9 million

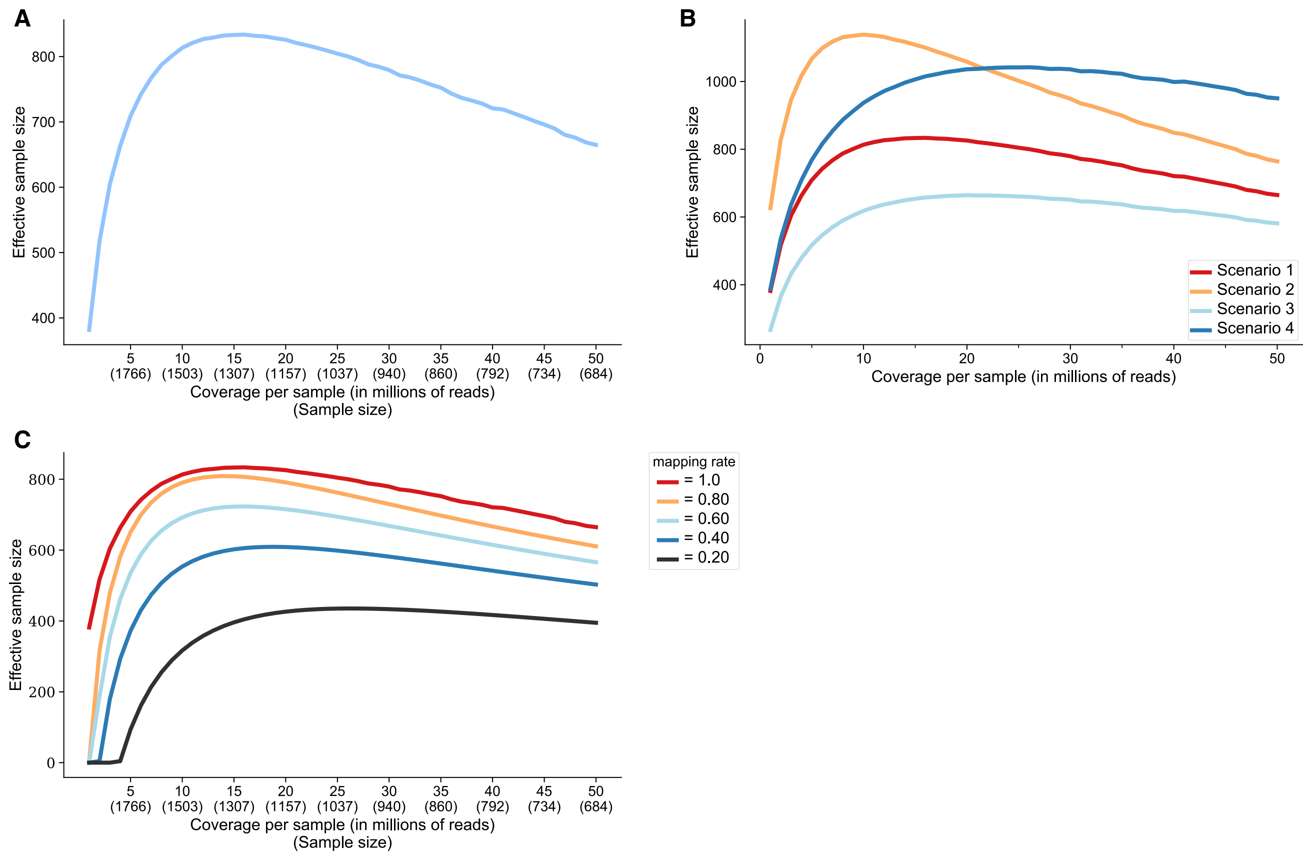


**Figure 4. Effective sample size under various budget parameters**
(A) Effective sample size in RNA-seq under a fixed budget ($300,000) as a function of the number of samples and the resulting coverage. Cost assumptions: $87 per library prep per sample, $1,790 per lane of sequencing (300 million reads), $53 per genotyped sample.
(B) Effective sample size in RNA-seq under a fixed budget ($300,000) as a function of the number of samples and the resulting coverage. Cost assumptions vary and are reflected in Table 4.
(C) Effective sample size under a fixed budget ($300,000) as a function of the number of samples and the results coverage. A global mapping rate parameter is used to simulate actual experimental conditions (materials and methods).

reads/sample, on average) under these assumptions is ~$293,000, just 47% of the cost of the GTEx experiment. The GTEx eQTL analysis reports 10,544 eGenes with a significant association, while using the lower-coverage RNA-seq leads to 7,587 eGenes with a significant association, 72% of what GTEx reports. If we assume that genotypes have already been measured in the cohort (such that $g = 0$), the cost of the lower-coverage RNA-seq experiment comes out to $215,000, while the GTEX experiment comes out to ~$585,000. This means that using just ~36% of the cost, lower-coverage RNA-seq has the power to detect ~72% of the eGenes with a significant association.

**Table 4. TWAS results for selected blood traits**

| Trait | Lower-coverage – n TWAS eGenes | GTEx – n TWAS eGenes |
|---|---|---|
| Mean corpuscular volume | 104 | 219 |
| Mean cell hemoglobin | 96 | 191 |
| Systemic lupus | 33 | 75 |
| All of the above | 143 | 311 |

The number of unique eGenes (columns 1 and 3) and GWAS SNPs (columns 2 and 4) significant (FDR $< 0.05$) in TWAS on eQTLs with significant heritability from analyses using lower-coverage RNA-seq (columns 1 and 2) and results from GTEx (columns 3 and 4).

## Discussion

In this work, we generate RNA-seq data at a lower coverage than typically used in eQTL studies (5.9 million reads/sample) and demonstrate how this approach boosts effective sample size per unit cost in an association study. To further validate this approach, we use synthetic RNA-seq data to show that the optimal level of coverage in an RNA-seq project for the purpose of identifying eQTL associations is lower than is commonly practiced.[11–14] Based on our findings, we recommend increasing sample size while lowering sequencing depth per sample to achieve optimal statistical power in association studies.

Our study is, in part, motivated by previous findings of whole-genome sequencing (WGS) studies benefiting from reduced coverage and increased sample sizes. We note that, although our application is similar, there

remains some key differences. Primarily, there exists a high variance in the degree to which transcripts are expressed, which is not easily predictable.[16] While we generally refer to experiment-wide coverage of an experiment, coverage differs across transcripts due to factors such as gene length and number of transcripts per gene. Consequently, the nature of RNA-seq data is such that lowering coverage of sequencing does not necessarily have a uniform effect on read sampling, which introduces an additional source of noise. It is important to explore the effects of reducing coverage in RNA-seq as the necessary level of coverage in WGS studies is generally dictated by the structural variant (SNP, indel, CNV) of interest, with a fairly predictable change in detection with reduced coverage. On the other hand, the necessary level of coverage in RNA-seq is related to its ability to detect lesser abundant transcripts, where the relationship between decreasing coverage and ability to quantify these transcripts is not understood as well.

We conclude with some notes, caveats, and future directions. First, synthetic RNA-seq via downsampling reads is potentially limited in several ways. These synthetic datasets of lower-coverage RNA-seq are created by uniformly sampling from real RNA-seq data with an average of 50 million reads mapped per sample. However, in practice, it is possible that sequencing biases are not captured by uniform sampling due to the different experimental setup compared with the dataset from which we sample.[24,32,39,45] In addition, these synthetic datasets are based on data obtained from fibroblast tissue with different transcriptomic profiles from whole blood, potentially influencing the sequencing depth required to detect associations with gene expression. Finally, this approach is optimized for eQTL discovery. Other mechanisms that are detected using RNA-seq, such as RNA splicing, have different mechanisms and will likely have different optimal coverages for detection. The fact that we identify different sets of eGenes depending on which gene expression measurements we consider (GTEx versus eQTLGen versus lower-coverage RNA-seq), shows that we need to increase cohort sizes in order to fully understand the connection between genetics and gene expression in blood. Furthermore, the results in Figure 4A (figure showing effective sample size at various coverages) indicate that even including 1,490 individuals under this fixed budget is not enough to achieve the optimal effective sample size. Current approaches are not sufficient to understand the full landscape of eQTLs in whole-blood tissue, even while only considering a single genetic ancestry group. We compare the eGenes identified by GTEx, eQTLGen, and the lower-coverage RNA-seq (Figure S12) and find that no single study is sufficient in capturing all of the associations in whole blood. We also see evidence of this in Figures 2D, S5, and S6, where the lower-coverage, moderate-coverage, and GTEx datasets do not detect nearly as many eGenes from the lowest quintile of genes by mean expression. Furthermore, as observed by the relatively low levels of overlap in colocalization and TWAS hits between GTEx and the lower-coverage sequencing, larger sample sizes are necessary to understand the roles of eQTLs with respect to GWAS. As observed in GWAS, much larger sample sizes, including far more ancestral diversity in these samples, will enable discovery of novel associations in transcriptomics. Including non-European populations and considering the temporal aspect of gene expression will help us gain a more complete understanding of the blood transcriptome landscape in the entire population.

## Materials and methods

### Cohort description

The samples included are from a study with individuals ascertained for bipolar disorder (BP). The cohort consists of 916 individuals with BP, 358 controls, and 216 relatives of the individuals with BP. Data were generated according to protocols approved by the respective local ethics committees: the Medical Ethical Review Board at University Medical Center Utrecht and the Institutional Review Board at University of California Los Angeles. Informed consent was obtained from all subjects.

### Connection between effect size and $R^2$

If $g$ is the genotype at the SNP that we are testing for associations, and $\beta$ is the effect size of that SNP when regressing on the true gene expression, $y$, and $\widehat{\beta}$ is the effect size of that SNP when regressing on the estimated gene expression, $\tilde{y}$. The relationship between $y$ and $\widehat{y}$ is as follows that $R^2 = corr(y, \widehat{y})$. It follows that the estimates of effect size for an SNP on the true gene expression, $\widehat{\beta}$, are related to the estimate of effect size for an SNP on the estimated gene expression, $\widehat{\tilde{\beta}}$ as $\widehat{\tilde{\beta}} = cov(g, \tilde{y}) = cov(g, Ry + \varepsilon) = cov(g, Ry) + cov(g, \varepsilon) = R\widehat{\beta}$, where $\varepsilon$ is a random variable with mean 0 and variance 1. The association test statistics at low coverage is $x_{ground} = Ncor^2(g, y)$, thus implying that the association statistic at low coverage is $x_{low-coverage} = Ncor^2(g, \tilde{y}) = N\widehat{\tilde{\beta}}^2 = N(R\widehat{\beta})^2 = R^2 * Ncor^2(g, y) = R^2 x_{ground}$, where $N$ is the number of samples included in the association study.

### Budget model

We modeled the cost of a large-scale bulk RNA-seq experiment based on parameters from two different library prep techniques: (1) TruSeq Stranded plus rRNA and GlobinZero and (2) TruSeq Stranded polyA selected, both from the UCLA Neuroscience Genomics core. Cost, or $B$, is a function of the following: $a$, the library preparation cost per sample; $b$, the target coverage of each sample (in millions of reads); $c$, the cost per lane (which contains $d$ million reads); $d$, the number of reads per sequencing lane (in millions); $g$, the cost of genotyping per sample; $e$, the cost of DNA and RNA extraction per sample; $N$, the number of samples in the association study; and $f$, any additional upfront or computational costs associated with analysis. Altogether, we model the budget as follows; $B = N * e + N * g + N * a + \frac{N * b * c}{d} + f$.

### Genotyping pipeline

Genotypes for the lower-coverage whole-blood samples were obtained from the following platforms: OmniExpressExome (N = 810), PSYCH (N = 523), and COEX (N = 163). Given that the SNP-genotype data for both the fibroblast and whole-blood samples came from numerous studies using various genotyping platforms, the number of overlapping SNPs across all platforms

was <150,000, prompting us to perform imputation separately for each genotyping platform (supplemental note). Genotypes were first filtered for Hardy-Weinberg equilibrium $p < 1.0 \times 10^{-6}$ for controls and $p < 1.0 \times 10^{-10}$ for cases, with minor allele frequency (MAF) > 0.01, and SNP-missingness < 0.05, leaving 148,612 typed SNPs[40]. Table S1 provides the number of typed and imputed SNPs per platform after quality control.

Genotypes were imputed using the 1000 Genomes Project phase 3 reference panel[41] by chromosome using RICOPILI v.1[42] separately per genotyping platform. These platform-specific genotypes were then subsequently merged after imputation, applying an individual-missingness threshold of 10% and SNP-missingness of 5% for post-merge quality control. We restricted to only autosomal SNPs due to sex chromosome dosage, as commonly done.[13] Imputation quality was assessed by filtering variants where genotype probability is >0.8 and INFO score is >0.1, resulting in 2,289,732 autosomal SNPs. The low final number of imputed SNPs stems from relatively disjoint starting sets of quality-controlled, typed genotypes per platform, leading to smaller sets of high-quality imputed variants that overlapped across platforms (with less than 5% SNP-missingness). Despite this, we were able to use over 15-fold more variants in the merged imputed set compared with the typed merged set. Then subsets of genotypes for the fibroblast-specific individuals, lower-coverage-specific individuals, and higher-coverage-specific individuals were extracted from the merged file set to be used in the eQTL analyses.

## Synthetic low-coverage RNA-seq

We use high-coverage RNA-seq (average of 50 million reads/sample, TruSeq Stranded polyA selected) from a set of 150 cell lines derived from human fibroblast cells. We assume this to be the ground truth of gene expression. We used seqtk to randomly downsample reads at various coverages, uniformly. We performed five iterations of downsampling at each level of coverage to account for potential variability in the sampling and sequencing errors.

## RNA-seq processing pipeline

We used FASTQC to visually inspect the read quality from the lower-coverage whole-blood RNA-seq (5.9 million reads/sample), the moderate-coverage whole-blood RNA-seq (13.9 million reads/sample), and the high-coverage fibroblast RNA-seq (50 million reads/sample). We then used kallisto to pseudoalign reads to the GRCh37 gencode transcriptome (v.33) and quantify estimates for transcript expression. We aggregated transcript counts to obtain gene-level read counts using scripts from the GTEx consortium.[13]

## *cis*-eQTL mapping

Excluding related individuals (pi_hat > 0.2) from the analysis, we perform *cis*-eQTL analysis mapping using FastQTL,[36] with a defined window of 1 Mb both up- and downstream of every gene's transcription start site (TSS), for sufficiently expressed genes (TPM > 0.1 in 20% of individuals). We run the eQTL analysis in permutation pass mode (1,000 permutations, and perform multiple testing corrections using the q value FDR procedure, correcting at 5% unless otherwise specified. We then restrict our associations to the top (or leading) SNP per eGene.

## Transcriptome-wide association study and colocalization

We used the FUSION framework[4] to perform the transcriptome-wide association study (TWAS) and subsequent colocalization[25] analysis. We computed single-best eQTL models for all eGenes detected in the lower-coverage dataset with the FUSION.compute_weights.R script. As this framework is intended for cis-loci, for each gene we restricted to SNPs within a window of 250 kb around the gene start and gene end position from the set of imputed genotypes. For the functional phenotypes (input through the –pheno flag), we used the gene-level TPMs generated by aggregating kallisto transcript expression estimates using scripts from GTEx.[13,33] Once the weights were generated, we input them in the FUSION.assoc_test.R script along with summary statistics from blood-related GWAS: mean corpuscular volume[3], mean cell hemoglobin[37], and systemic lupus erythematosus[38]; the 1000 Genomes LD panel for European ancestries was used as the reference. Colocalization was performed on those gene-trait associations that had p values less than 0.05 (–coloc_P 0.05 flag). This pipeline was then repeated using the GTEx V8 whole-blood gene expression (using the GTEx pipeline) and corresponding SNP-genotypes from 668 unrelated donors.

## Covariates

For eQTL analyses conducted using the moderate-coverage whole-blood and synthetic data derived from fibroblasts, we include the top three genotype principal components and top 50 gene expression principal components, calculated separately for each synthetic dataset. For eQTL analyses conducted using the lower-coverage whole blood, we include the top 10 genotype PCs (to account for the differences across the multiple genotyping platforms used to genotype samples in this cohort), and the top 50 expression PCs. In eQTL analyses using synthetic data we also include sex and several cell line technical covariates (passage number and growth rate). In eQTL analyses using moderate-coverage whole blood, we include sex, disease status, and age. In eQTL analyses using lower-coverage whole blood, we include sex, disease status, genotyping platform, and several technical covariates regarding the tissue samples (RIN and concentration).

## Cell-type proportion estimation

We estimate the proportion of cell types of both the lower-coverage and moderate-coverage bulk whole-blood RNA-seq datasets using CIBERSORTx[43] with batch correction applied and LM22 signature matrix as the reference gene expression profile. The LM22 signature matrix uses 547 genes to distinguish between 22 human hematopoietic cell phenotypes.

## $R^2$ adjustment

To account for the variability in mapping rate across different library prep techniques and different tissue types,[44,45] we look at the mean $R^2$ at the expected coverage, which is calculated as: expected coverage = target coverage * estimated mapping rate. Using mean $R^2$ values from comparing lower-coverage synthetic RNA-seq to moderate-coverage RNA-seq real data, we fit a log curve to estimate the adjusted mean $R^2$ ($R^2_{adj}$) at the expected coverage.

## Effective sample size

Under a fixed budget setting, we calculate effective sample size ($N_{eff}$) for a given coverage using the adjusted mean $R^2$ ($R^2_{adj}$) and the number of samples included at a given coverage level (N) $N_{eff} = R^2_{adj} * N$.

## Data and code availability

The lower-coverage RNA-seq and the corresponding genotypes generated and analyzed during this study will be deposited in dbGAP (accession number phs002856.v1).

## Supplemental information

Supplemental information can be found online at https://doi.org/10.1016/j.xhgg.2022.1001)03.

## Acknowledgments

We thank the study subjects for their willingness to provide specimens and clinical data. We thank Yi Ding, Kathryn Burch, Ruthie Johnson, Arjun Bhattacharya, and Malika Freund for meaningful discussion in helping make this work possible. T.S. was supported by the National Institute of Neurological Disorders and Stroke of the National Institutes of Health under award no. T32NS048004. T.B. was supported by the NIH (grant no. 5T32HG002536-19). This research was supported by the National Institute of Mental Health of the National Institutes of Health under award no. 5R01MH115676-04. The content is solely the responsibility of the authors and does not necessarily represent the official views of the National Institutes of Health.

## Declaration of interests

The authors declare no competing interests.

Received: January 20, 2022
Accepted: March 29, 2022

## Web resources

seqtk, https://github.com/lh3/seqtk

GTEx consortium, https://github.com/broadinstitute/gtex-pipeline

RNA-Seq Coverage Calculator, https://tomschwarz.shinyapps.io/RNASeqCoverageCalculator/

## References

1. Montgomery, S.B., Sammeth, M., Gutierrez-Arcelus, M., Lach, R.P., Ingle, C., Nisbett, J., Guigo, R., and Dermitzakis, E.T. (2010). Transcriptome genetics using second generation sequencing in a Caucasian population. Nature *464*, 773–777.
2. Pickrell, J.K., Marioni, J.C., Pai, A.A., Degner, J.F., Engelhardt, B.E., Nkadori, E., Veyrieras, J.B., Stephens, M., Gilad, Y., and Pritchard, J.K. (2010). Understanding mechanisms underlying human gene expression variation with RNA sequencing. Nature *464*, 768–772.
3. Mancuso, N., Shi, H., Goddard, P., Kichaev, G., Gusev, A., and Pasaniuc, B. (2017). Integrating gene expression with summary association statistics to identify genes associated with 30 complex traits. Am. J. Hum. Genet. *100*, 473–487.
4. Gusev, A., Ko, A., Shi, H., Bhatia, G., Chung, W., Penninx, B.W., Jansen, R., de Geus, E.J., Boomsma, D.I., Wright, F.A., et al. (2016). Integrative approaches for large-scale transcriptome-wide association studies. Nat. Genet. *48*, 245–252.
5. Gamazon, E.R., Wheeler, H.E., Shah, K.P., Mozaffari, S.V., Aquino-Michaels, K., Carroll, R.J., Eyler, A.E., Denny, J.C., GTEx Consortium, Nicolae, D.L., Cox, N.J., and Im, H.K. (2015). A gene-based association method for mapping traits using reference transcriptome data. Nat. Genet. *47*, 1091–1098.
6. Zhernakova, D.V., Deelen, P., Vermaat, M., van Iterson, M., van Galen, M., Arindrarto, W., van 't Hof, P., Mei, H., van Dijk, F., Westra, H.J., et al. (2017). Identification of context-dependent expression quantitative trait loci in whole blood. Nat. Genet. *49*, 139–145.
7. Wang, Z., Gerstein, M., and Snyder, M. (2009). RNA-Seq: a revolutionary tool for transcriptomics. Nat. Rev. Genet. *10*, 57–63.
8. Rockman, M.V., and Kruglyak, L. (2006). Genetics of global gene expression. Nat. Rev. Genet. *7*, 862–872.
9. Gallagher, M.D., and Chen-Plotkin, A.S. (2018). The post-GWAS era: from association to function. Am. J. Hum. Genet. *102*, 717–730.
10. Võsa, U., Claringbould, A., Westra, H.J., Bonder, M.J., Deelen, P., Zeng, B., Kirsten, H., Saha, A., Kreuzhuber, R., Yazar, S., et al. (2021). Large-scale *cis*- and *trans*-eQTL analyses identify thousands of genetic loci and polygenic scores that regulate blood gene expression. Nat. Genet. *53*, 1300–1310. https://doi.org/10.1038/s41588-021-00913-z.
11. Hoffman, G.E., Bendl, J., Voloudakis, G., Montgomery, K.S., Sloofman, L., Wang, Y.C., Shah, H.R., Hauberg, M.E., Johnson, J.S., Girdhar, K., et al. (2019). CommonMind consortium provides transcriptomic and epigenomic data for schizophrenia and bipolar disorder. Sci. Data *6*, 180.
12. Franzén, O., Ermel, R., Cohain, A., Akers, N.K., Di Narzo, A., Talukdar, H.A., Foroughi-Asl, H., Giambartolomei, C., Fullard, J.F., Sukhavasi, K., et al. (2016). Cardiometabolic risk loci share downstream cis- and trans-gene regulation across tissues and diseases. Science *353*, 827–830.
13. GTEx Consortium (2020). The GTEx Consortium atlas of genetic regulatory effects across human tissues. Science *369*, 1318–1330.
14. Lepik, K., Annilo, T., Kukuškina, V., eQTLGen Consortium, Kisand, K., Kutalik, Z., Peterson, P., and Peterson, H. (2017). C-reactive protein upregulates the whole blood expression of *CD59* - an integrative analysis. PLoS Comput. Biol. *13*, e1005766. https://doi.org/10.1371/journal.pcbi.1005766.
15. Pasaniuc, B., Rohland, N., McLaren, P.J., Garimella, K., Zaitlen, N., Li, H., Gupta, N., Neale, B.M., Daly, M.J., Sklar, P., et al. (2012). Extremely low-coverage sequencing and imputation increases power for genome-wide association studies. Nat. Genet. *44*, 631–635.
16. Sims, D., Sudbery, I., Ilott, N.E., Heger, A., and Ponting, C.P. (2014). Sequencing depth and coverage: key considerations in genomic analyses. Nat. Rev. Genet. *15*, 121–132.
17. CONVERGE consortium (2015). Sparse whole-genome sequencing identifies two loci for major depressive disorder. Nature *523*, 588–591.
18. Homburger, J.R., Neben, C.L., Mishne, G., Zhou, A.Y., Kathiresan, S., and Khera, A.V. (2019). Low coverage whole genome sequencing enables accurate assessment of common variants and calculation of genome-wide polygenic scores. Genome Med. *11*, 74.
19. Rubinacci, S., Ribeiro, D.M., Hofmeister, R.J., and Delaneau, O. (2021). Efficient phasing and imputation of low-coverage

sequencing data using large reference panels. Nat. Genet. *53*, 412.
20. Baccarella, A., Williams, C.R., Parrish, J.Z., and Kim, C.C. (2018). Empirical assessment of the impact of sample number and read depth on RNA-Seq analysis workflow performance. BMC Bioinf. *19*, 423.
21. Pritchard, J.K., and Przeworski, M. (2001). Linkage disequilibrium in humans: models and data. Am. J. Hum. Genet. *69*, 1–14.
22. Tarazona, S., García-Alcalde, F., Dopazo, J., Ferrer, A., and Conesa, A. (2011). Differential expression in RNA-seq: a matter of depth. Genome Res. *21*, 2213–2223.
23. Robinson, D.G., and Storey, J.D. (2014). subSeq: determining appropriate sequencing depth through efficient read subsampling. Bioinformatics *30*, 3424–3426.
24. Williams, A.G., Thomas, S., Wyman, S.K., and Holloway, A.K. (2014). RNA-seq data: challenges in and recommendations for experimental design and analysis. Curr. Protoc. Hum. Genet. *83*, 11.13.1–20.
25. Giambartolomei, C., Vukcevic, D., Schadt, E.E., Franke, L., Hingorani, A.D., Wallace, C., and Plagnol, V. (2014). Bayesian test for colocalisation between pairs of genetic association studies using summary statistics. PLoS Genet. *10*, e1004383. https://doi.org/10.1371/journal.pgen.1004383.
26. Lloyd-Jones, L.R., Holloway, A., McRae, A., Yang, J., Small, K., Zhao, J., Zeng, B., Bakshi, A., Metspalu, A., Dermitzakis, M., et al. (2017). The genetic architecture of gene expression in peripheral blood. Am. J. Hum. Genet. *100*, 228–237. https://doi.org/10.1016/j.ajhg.2016.12.008.
27. GTEx Consortium (2017). Genetic effects on gene expression across human tissues. Nature *550*, 204–213. https://doi.org/10.1038/nature24277.
28. Yao, D.W., O'Connor, L.J., Price, A.L., and Gusev, A. (2020). Quantifying genetic effects on disease mediated by assayed gene expression levels. Nat. Genet. *52*, 626–633. https://doi.org/10.1038/s41588-020-0625-2.
29. Mu, Z., Wei, W., Fair, B., Miao, J., Zhu, P., and Li, Y.I. (2021). The impact of cell type and context-dependent regulatory variants on human immune traits. Genome Biol. *22*, 122. https://doi.org/10.1186/s13059-021-02334-x.
30. Lachmann, A., Torre, D., Keenan, A.B., Jagodnik, K.M., Lee, H.J., Wang, L., Silverstein, M.C., and Ma'ayan, A. (2018). Massive mining of publicly available RNA-seq data from human and mouse. Nat. Commun. *9*, 1366. https://doi.org/10.1038/s41467-018-03751-6.
31. Krebs, C.E., Ori, A.P.S., Vreeker, A., Wu, T., Cantor, R.M., Boks, M.P.M., Kahn, R.S., Olde Loohuis, L.M., and Ophoff, R.A. (2020). Whole blood transcriptome analysis in bipolar disorder reveals strong lithium effect. Psychol. Med. *50*, 2575–2586.
32. Frazee, A.C., Jaffe, A.E., Langmead, B., and Leek, J.T. (2015). Polyester: simulating RNA-seq datasets with differential transcript expression. Bioinformatics *31*, 2778–2784.
33. Bray, N.L., Pimentel, H., Melsted, P., and Pachter, L. (2016). Near-optimal probabilistic RNA-seq quantification. Nat. Biotechnol. *34*, 525–527.
34. Liu, Y., Ferguson, J.F., Xue, C., Silverman, I.M., Gregory, B., Reilly, M.P., and Li, M. (2013). Evaluating the impact of sequencing depth on transcriptome profiling in human adipose. PLoS One *8*, e66883.
35. Mandric, I., Schwarz, T., Majumdar, A., Hou, K., Briscoe, L., Perez, R., Subramaniam, M., Hafemeister, C., Satija, R., Ye, C.J., et al. (2020). Optimized design of single-cell RNA sequencing experiments for cell-type-specific eQTL analysis. Nat. Commun. *11*, 5504.
36. Ongen, H., Buil, A., Brown, A.A., Dermitzakis, E.T., and Delaneau, O. (2016). Fast and efficient QTL mapper for thousands of molecular phenotypes. Bioinformatics *32*, 1479–1485.
37. van der Harst, P., Zhang, W., Mateo Leach, I., Rendon, A., Verweij, N., Sehmi, J., Paul, D.S., Elling, U., Allayee, H., Li, X., et al. (2012). Seventy-five genetic loci influencing the human red blood cell. Nature *492*, 369–375.
38. Bentham, J., Morris, D.L., Graham, D.S.C., Pinder, C.L., Tombleson, P., Behrens, T.W., Martín, J., Fairfax, B.P., Knight, J.C., Chen, L., et al. (2015). Genetic association analyses implicate aberrant regulation of innate and adaptive immunity genes in the pathogenesis of systemic lupus erythematosus. Nat. Genet. *47*, 1457–1464.
39. Marioni, J.C., Mason, C.E., Mane, S.M., Stephens, M., and Gilad, Y. (2008). RNA-seq: an assessment of technical reproducibility and comparison with gene expression arrays. Genome Res. *18*, 1509–1517.
40. Purcell, S., Neale, B., Todd-Brown, K., Thomas, L., Ferreira, M.A., Bender, D., Maller, J., Sklar, P., de Bakker, P.I., Daly, M.J., and Sham, P.C. (2007). PLINK: a tool set for whole-genome association and population-based linkage analyses. Am. J. Hum. Genet. *81*, 559–575.
41. The 1000 Genomes Project Consortium, Auton, A., Brooks, L.D., Durbin, R.M., Garrison, E.P., Kang, H.M., Korbel, J.O., Marchini, J.L., McCarthy, S., McVean, G.A., and Abecasis, G.R. (2015). A global reference for human genetic variation. Nature *526*, 68–74.
42. Lam, M., Awasthi, S., Watson, H.J., Goldstein, J., Panagiotaropoulou, G., Trubetskoy, V., Karlsson, R., Frei, O., Fan, C.C., De Witte, W., et al. (2019). RICOPILI: rapid imputation for COnsortias PIpeLIne. Bioinformatics *36*, 930–933.
43. Newman, A.M., Steen, C.B., Liu, C.L., Gentles, A.J., Chaudhuri, A.A., Scherer, F., Khodadoust, M.S., Esfahani, M.S., Luca, B.A., Steiner, D., et al. (2019). Determining cell type abundance and expression from bulk tissues with digital cytometry. Nat. Biotechnol. *37*, 773–782.
44. Aguet, F., and Muñoz Aguirre, M. (2017). Genetic effects on gene expression across human tissues. Nature *550*, 204–213.
45. Daley, T., and Smith, A.D. (2013). Predicting the molecular complexity of sequencing libraries. Nat. Methods *10*, 325–327. https://doi.org/10.1038/nmeth.2375.

HGGA, Volume *3*

## Supplemental information

# Powerful eQTL mapping through low-coverage RNA sequencing

**Tommer Schwarz, Toni Boltz, Kangcheng Hou, Merel Bot, Chenda Duan, Loes Olde Loohuis, Marco P. Boks, René S. Kahn, Roel A. Ophoff, and Bogdan Pasaniuc**

## SUPPLEMENTARY FIGURES

**S1A**:

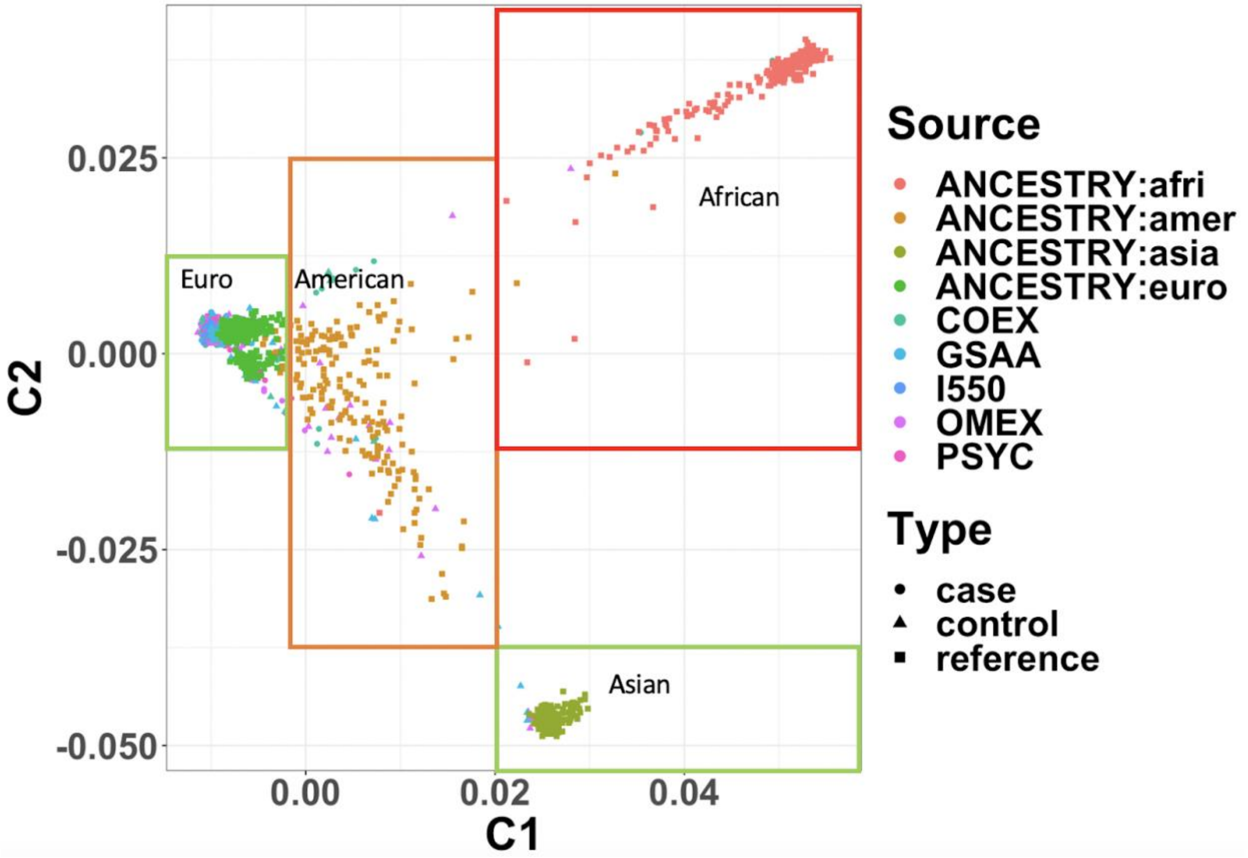


**S1B**:

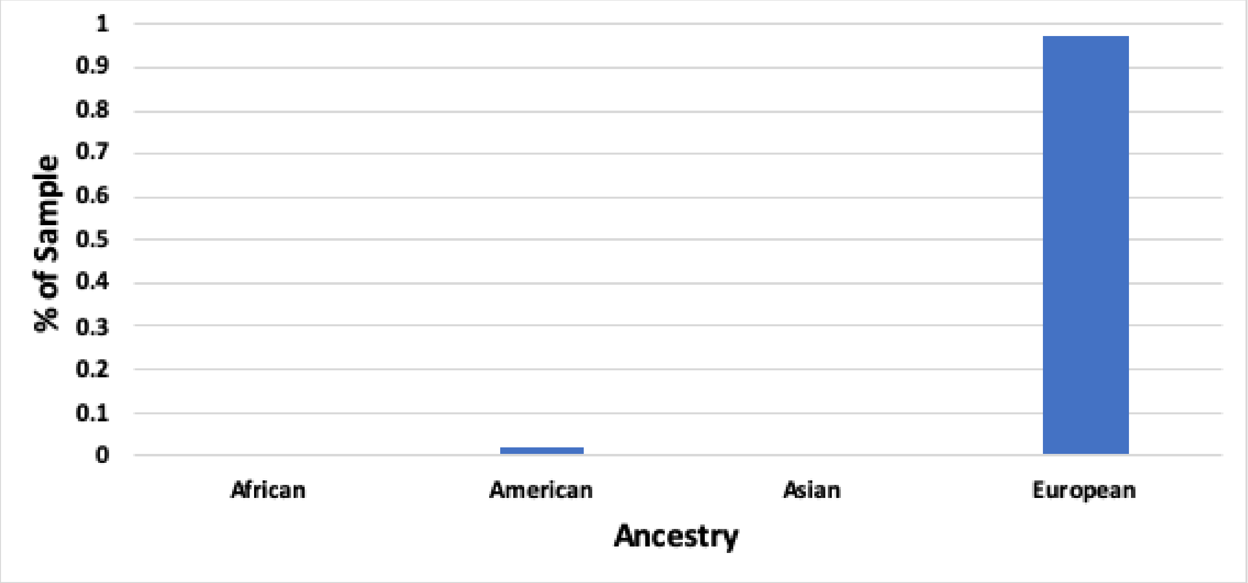

S1C:

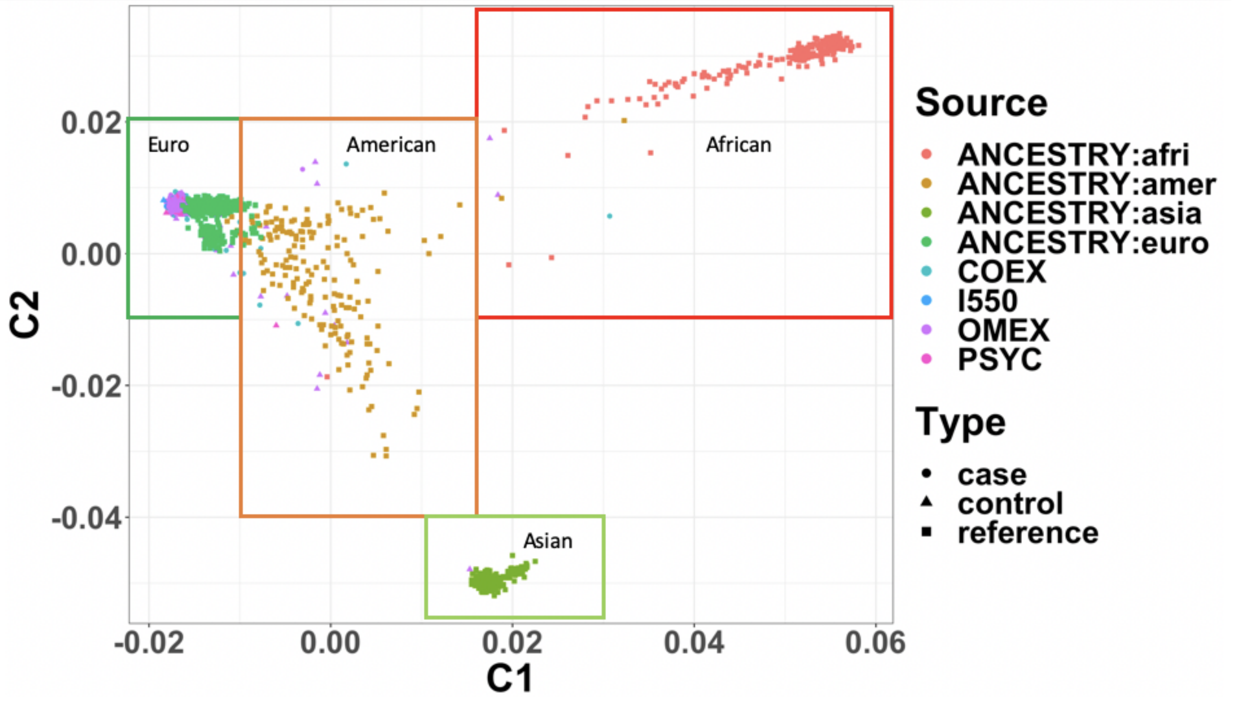


S1D:

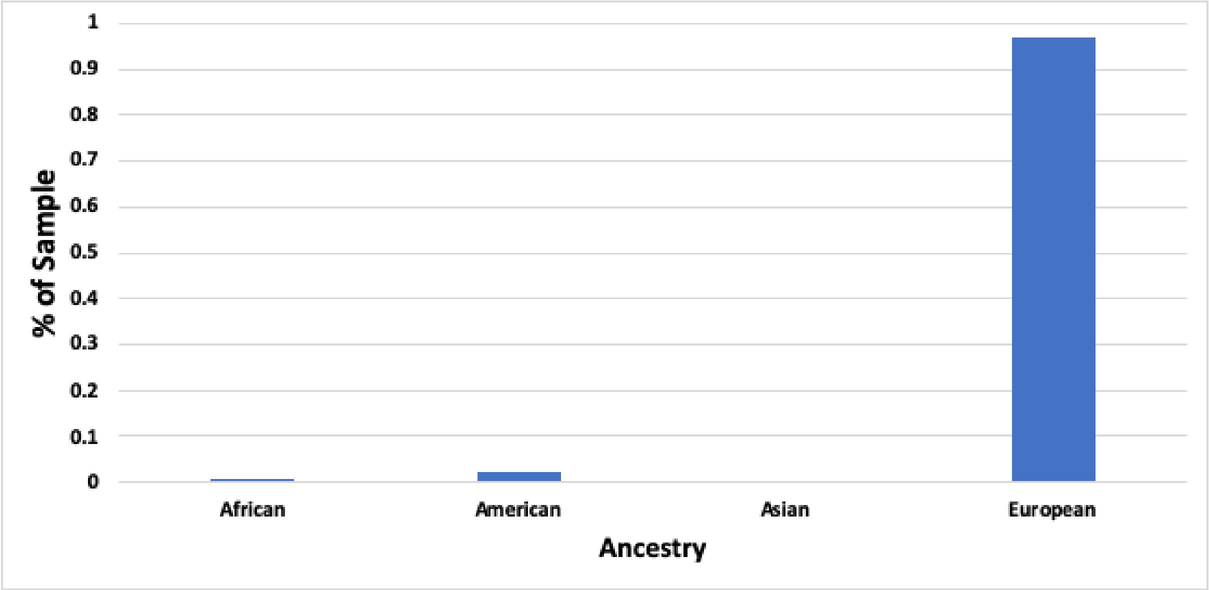


**Figure S1:** *Distribution of ancestry among samples.* **(S1A)** Genotype PC1 and PC2 are projected onto PCs from 1000 Genomes Project. Points labeled with "ANCESTRY" are from 1000 Genomes Project, remaining points designate the specific genotyping platform used in our cohort. Boxes are drawn around the centers to show where samples from the n = 2000 / 5.9M reads/sample cohort lie. **(S1B)** A barplot showing the distribution of ancestry observed in the n = 2000 / 5.9M reads/sample cohort, according to the MDS plot. Note that only the 1963 samples that pass genotype QC thresholds are included here. Exact numbers of samples per ancestry group are: African - 4, American - 34, Asian - 9, European – 1916. **(S1C)**: Genotype PC1 and PC2 are projected onto PCs from 1000 Genomes Project. Points labeled with "ANCESTRY" are from 1000

Genomes Project, remaining points designate the specific genotyping platform used in our cohort. Boxes are drawn around the centers to show where samples from the n = 759 / 13.9M reads/sample cohort lie. **(S1D)** A barplot showing the distribution of ancestry observed in the n = 759 / 13.9M reads/sample cohort, according to the MDS plot. Exact numbers of samples per ancestry group are: African - 4, American - 19, Asian - 1, European – 735.

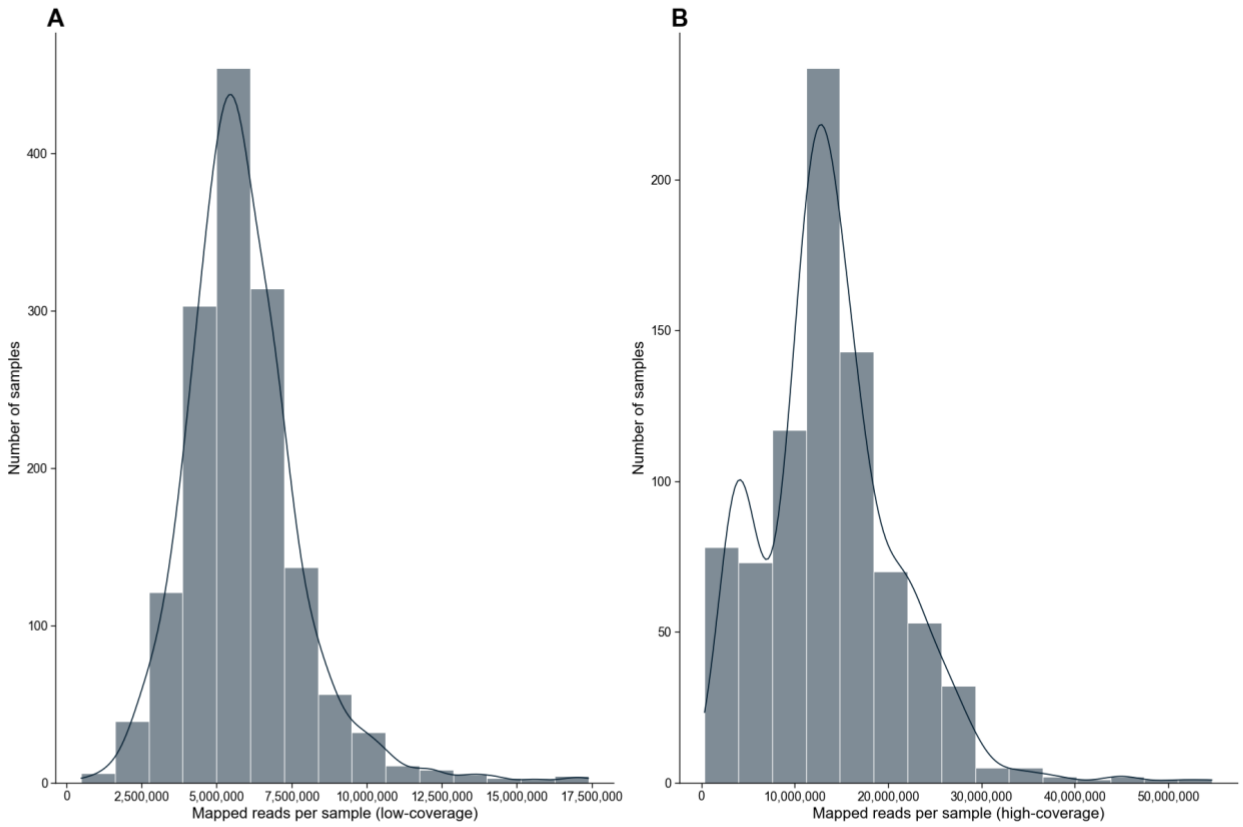


**Figure S2:** *Number of pseudoaligned reads per sample for low-coverage and high-coverage experiments*. **(S2A)** In real data, a histogram showing the number of reads mapped to genes (or in kallisto terms: number of reads for which transcriptome successfully mapped), per sample. **(S2B)** In real data, a histogram showing the number of reads mapped to genes (or in kallisto terms: number of reads for which transcriptome successfully mapped), per sample.

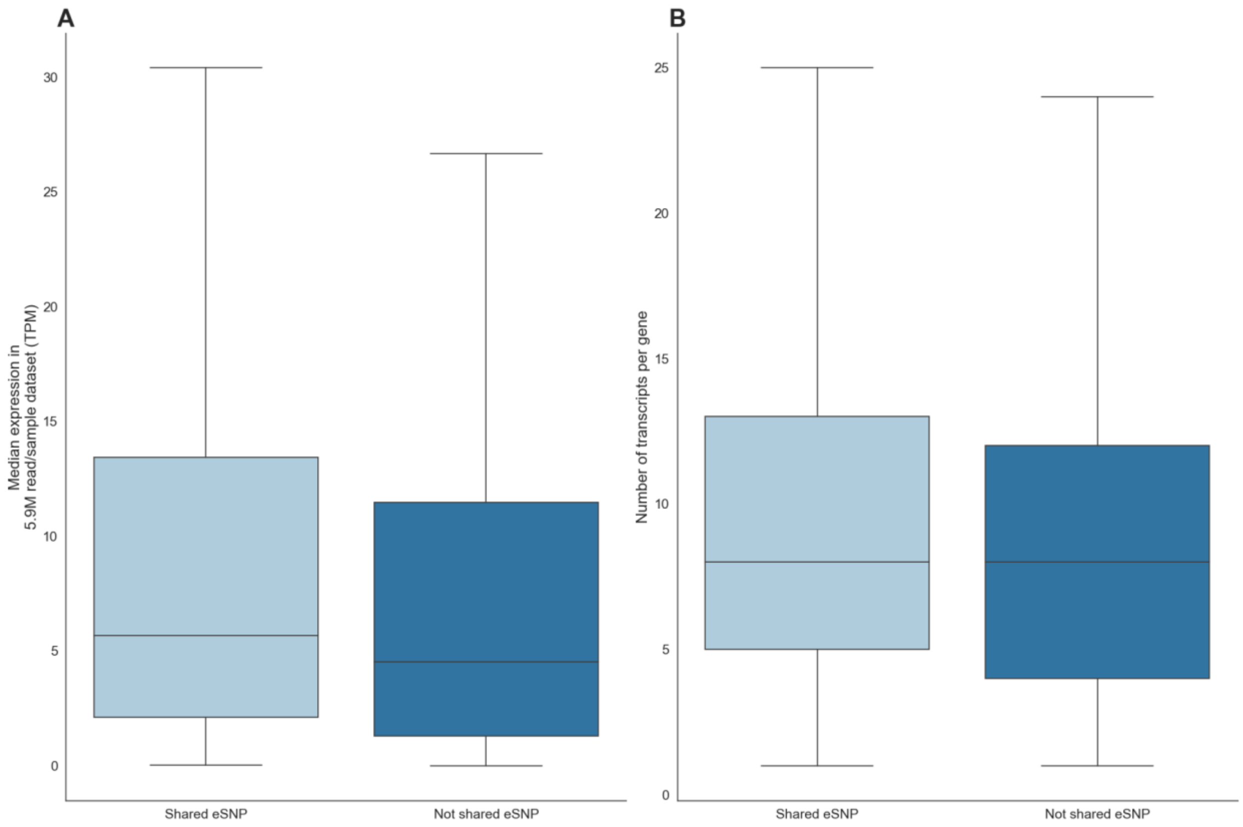


**Figure S3:** *Characteristics of eGenes that do/do not share the same eSNP between lower-coverage and moderate-coverage RNA-Seq.* **(S3A)** eGenes with the same eSNP have an average expression of 15.6 TPM (sd = 51.1) while eGenes that do not share the same eSNP have an expression of 14.2 TPM (sd = 40.8), (p = 0.03). (**S3B)** The average number of isoforms for eGenes that shared an eSNP was 10.0 (sd = 8.0), while it is only 9.6 (sd = 7.9) for those eGenes that do not share an eSNP (p = 0.03).

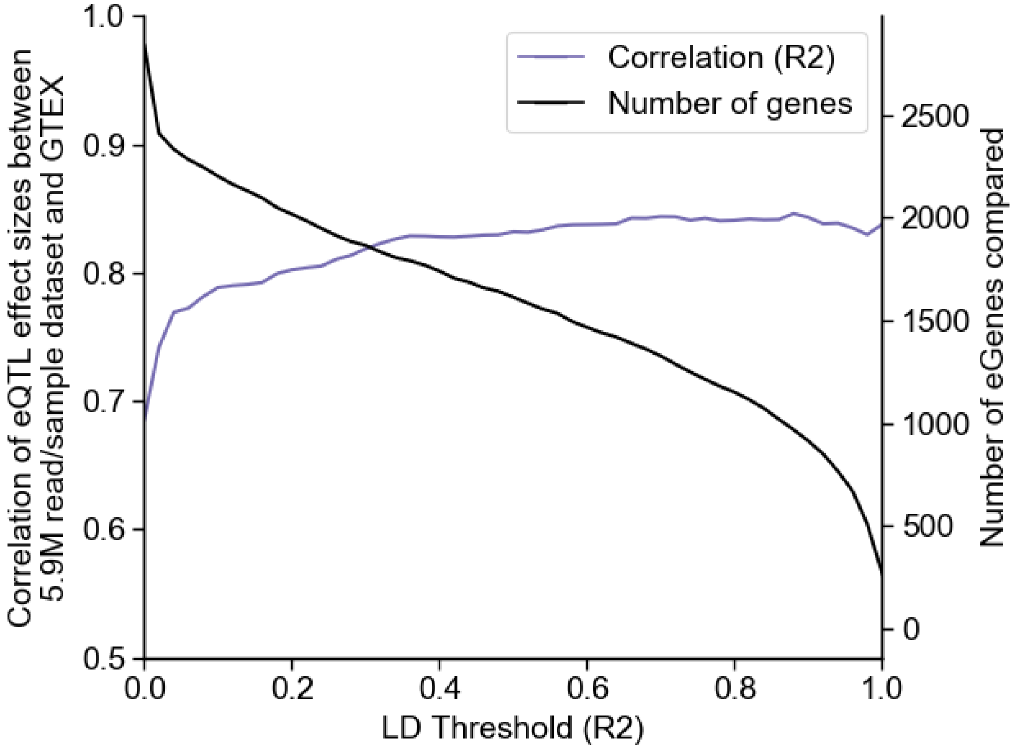


**Figure S4:** *Correlation of effect sizes between eQTL analyses low-coverage and GTEX with respect to LD-threshold between eSNPs.* **(S4)** On the x-axis, we show the LD threshold used to restrict our comparisons of effect sizes between eQTLs found in the low-coverage dataset and GTEX. Only eGenes with the same eSNP, or eSNPs with an LD above the threshold are used. On the left-hand y-axis, we show the correlation of the effect sizes. On the right-hand y-axis, we show the number of eGenes compared under the given LD threshold.

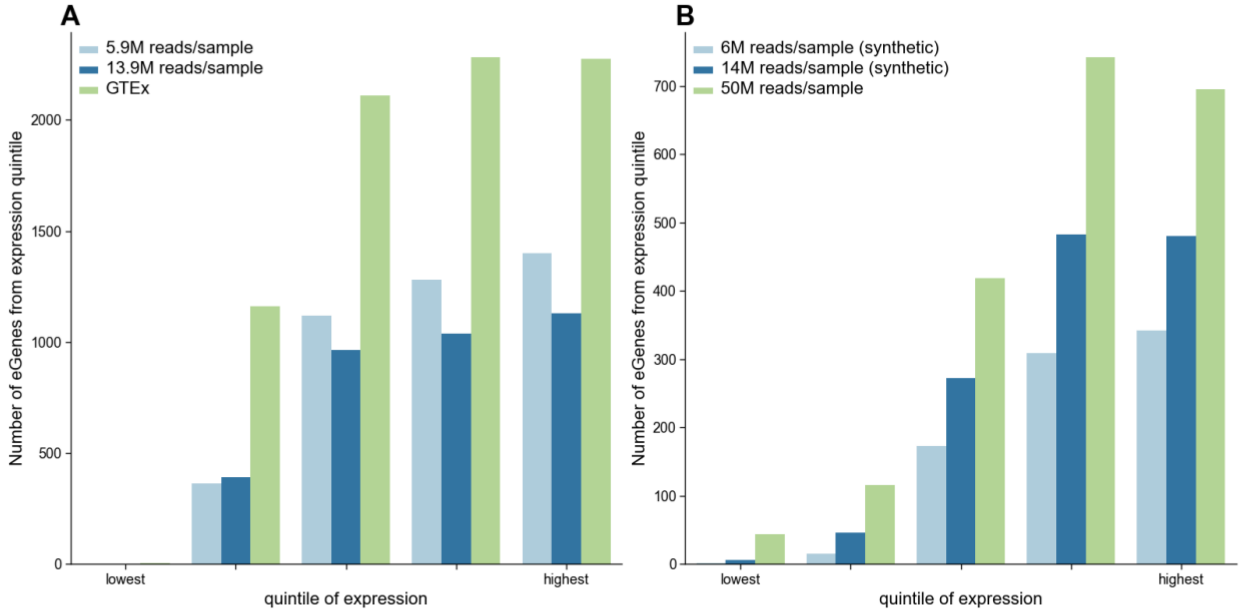


**Figure S5:** *Number of eGenes per mean expression quintile across datasets.* **(S5A)** Stratifying the 19175 protein coding genes reported in GTEx into quintile groups by mean expression, on the x-axis, we show the quintile groups by increasing mean expression. On the y-axis, we show the number of eGenes found in the (1) low-coverage, (2) high-coverage, and (3) GTEx experiments, in each of these quintile groups. **(S5B)** Stratifying the 24206 protein coding genes discovered in the high-coverage fibroblast dataset into quintile groups by mean expression, on the x-axis, we show the quntile groups by increasing mean expression. On the y-axis, we show the number of eGenes found in the (1) synthetic RNA-Seq at 6M reads/sample, (2) synthetic RNA-Seq at 14M reads/sample, and (3) RNA-Seq data at 50M reads/sample, in each of these quintile groups.

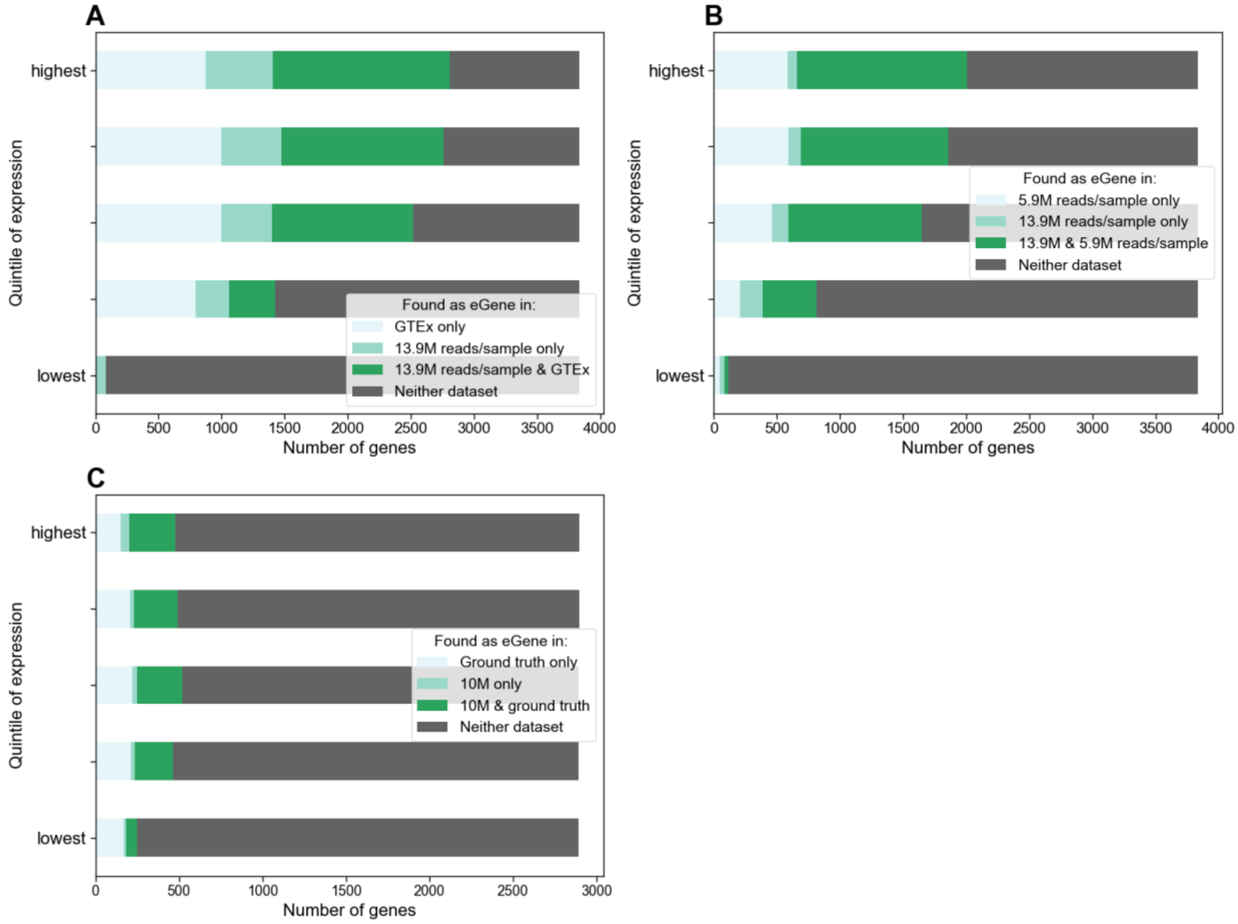


**Figure S6:** *eGene concordance by quintile in moderate-coverage RNA-Seq and GTEx:* **(S6A):** The overlap in eGenes between moderate-coverage RNA-Seq and GTEX, stratified into quintiles by the mean expression level observed in GTEX. **(S6B):** The overlap in eGenes between moderate-coverage RNA-Seq and lower-coverage RNA-Seq, stratified into quintiles by the mean expression level observed in GTEX. **(S6C):** The overlap in eGenes between high-coverage "ground-truth" RNA-Seq and a 10M read/sample synthetic dataset, stratified into quintiles by the mean expression level observed in the high-coverage RNA-Seq

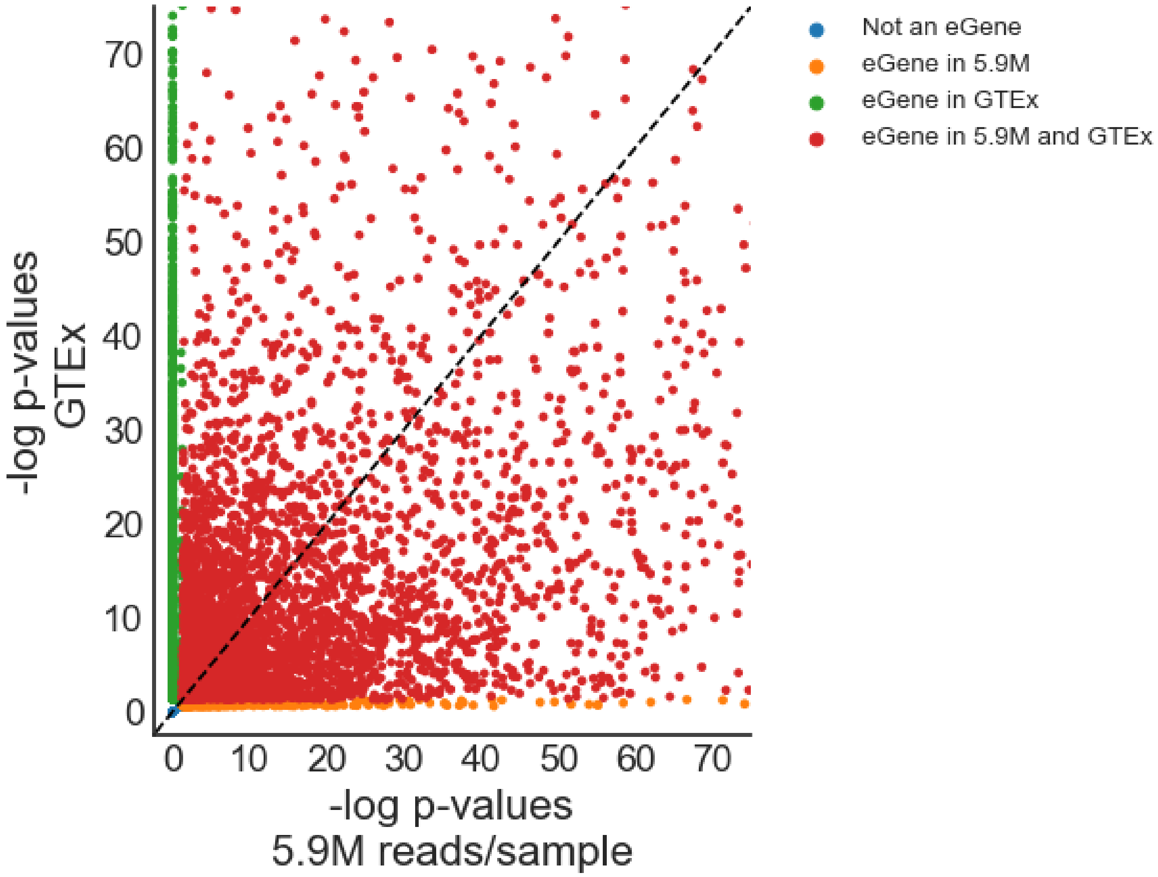


**Figure S7:** *Real data p-value comparison scatterplot with GTEX*. **(S3A)** Using the 12,496 protein-coding genes included both in GTEX and the low-coverage datasets, on the x-axis, we show the -log p-values for leading SNP eQTL associations in the low-coverage dataset. On the y-axis, we show the -log p-values for leading SNP eQTL associations in the GTEX dataset.

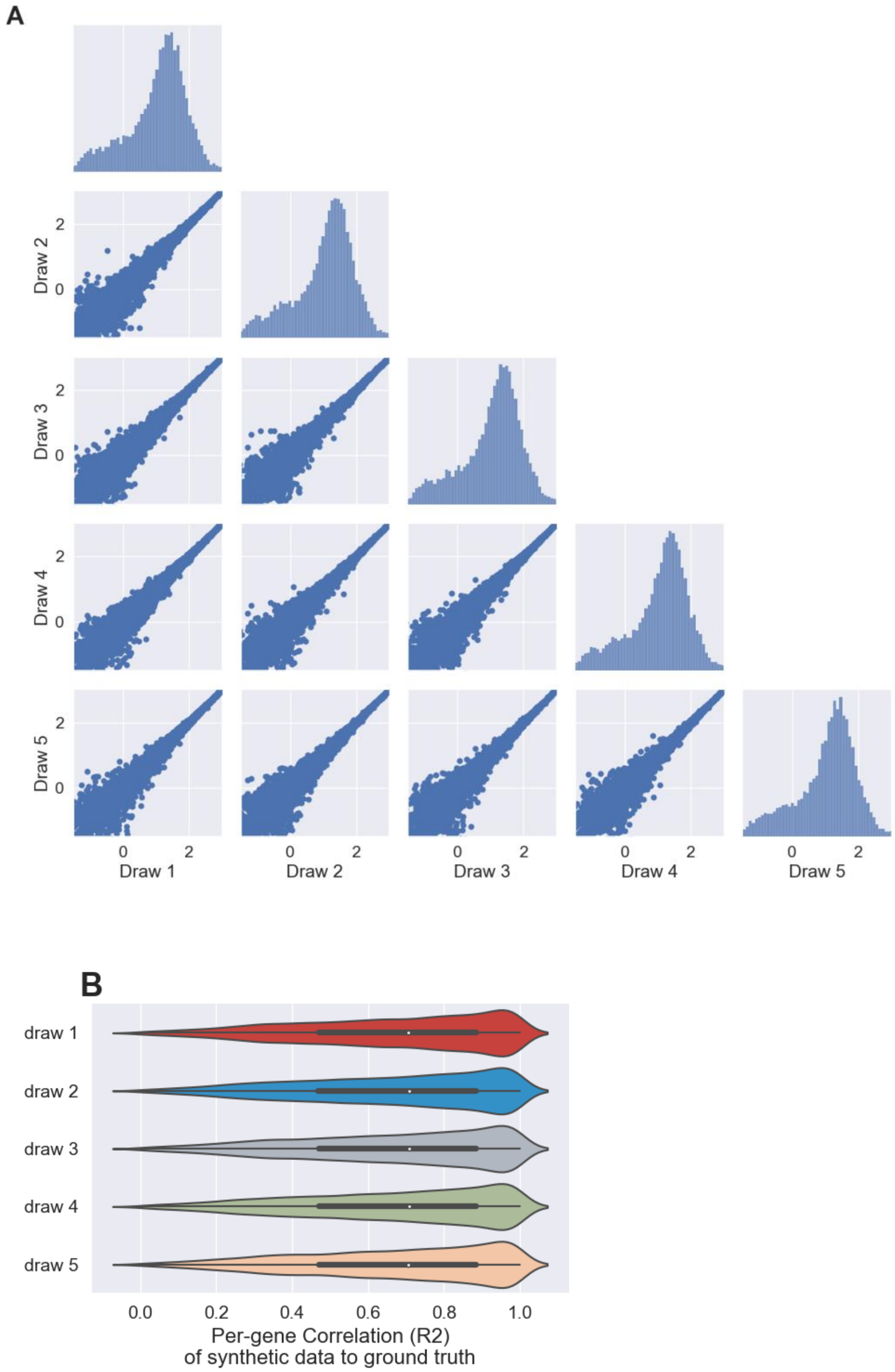


**Figure S8:** V*ariability in correlations in synthetic data*. **(S8A)** For synthetic data corresponding to one sample, a comparison of estimated log TPM values between five different uniform sampling

draws at 10 million reads/sample, for 14,948 protein-coding genes. **(S8B)** For 14,948 protein-coding genes estimated across five different uniform sampling draws at 10 million reads/sample, we compare the distribution of correlation ($R^2$) between the estimated expression of the samples and the ground truth gene expression.

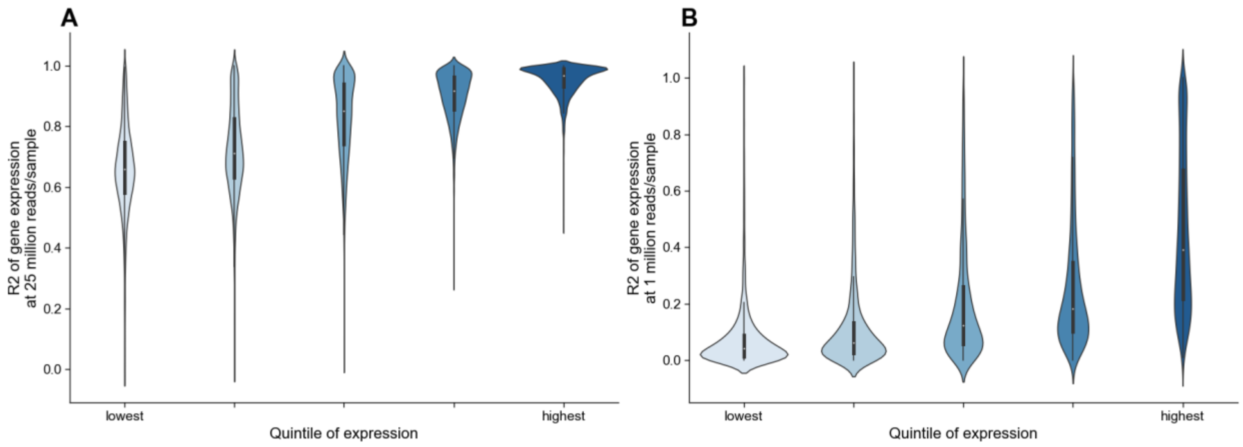


**Figure S9:** *Variability of correlations as a function of average expression in a given gene* **(S9A)** Gene expression accuracy using data simulated with 1 million reads/sample, as a function of relative gene expression observed in actual RNA-Seq data with 50 million reads/sample. 23,043 genes (with average expression < 0.1 TPM) are divided into five ascending quintiles of expression based on their average expression in 155 samples. **(S9B)** Gene expression accuracy using data simulated with 1 million reads/sample, as a function of relative gene expression observed in actual RNA-Seq data with 50 million reads/sample. 23,043 genes (with average expression < 0.1 TPM) are divided into five ascending quintiles of expression based on their average expression in 155 samples.

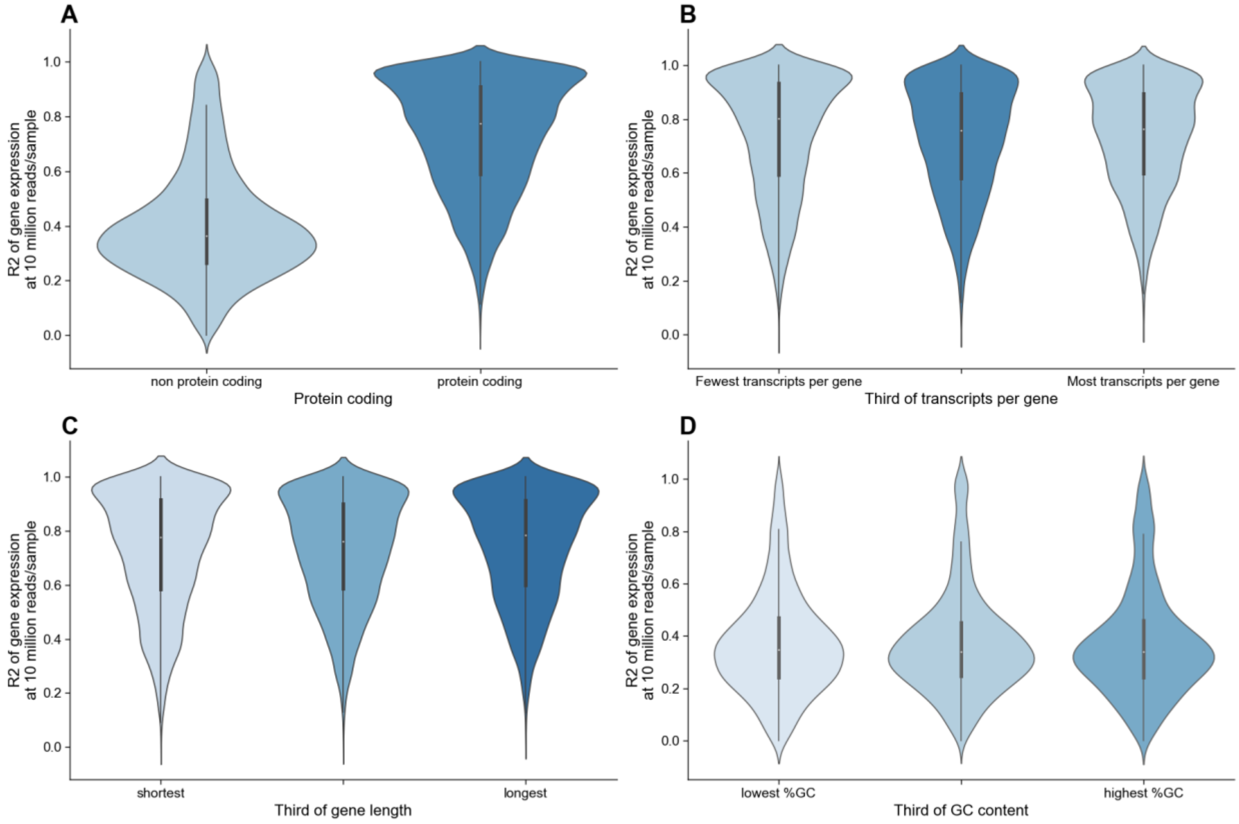


**Figure S10:** *Variability of correlation as a function of various gene characteristics*. **(S10A)** Gene expression estimation accuracy simulated at 10 million reads/sample as a function of whether a gene codes for a protein. 24,093 genes (with average expression < 0.1 TPM) are divided into two groups. **(S10B)** Gene expression estimation accuracy simulated at 10 million reads/sample as a function of how many transcripts each gene has. 23,540 genes (with average expression < 0.1 TPM) are divided into three ascending groups based on the number of transcripts contained in each gene. **(S10C)** Gene expression estimation accuracy simulated at 10 million reads/sample as a function of relative gene length. 14,484 genes (with average expression < 0.1 TPM, protein coding) are divided into three groups based on the length of each gene. **(S10D)** Gene expression accuracy as a function of relative GC content. 5,771 genes (with average expression < 0.1 TPM and GC content reported) are divided into three groups based on the length of each gene.

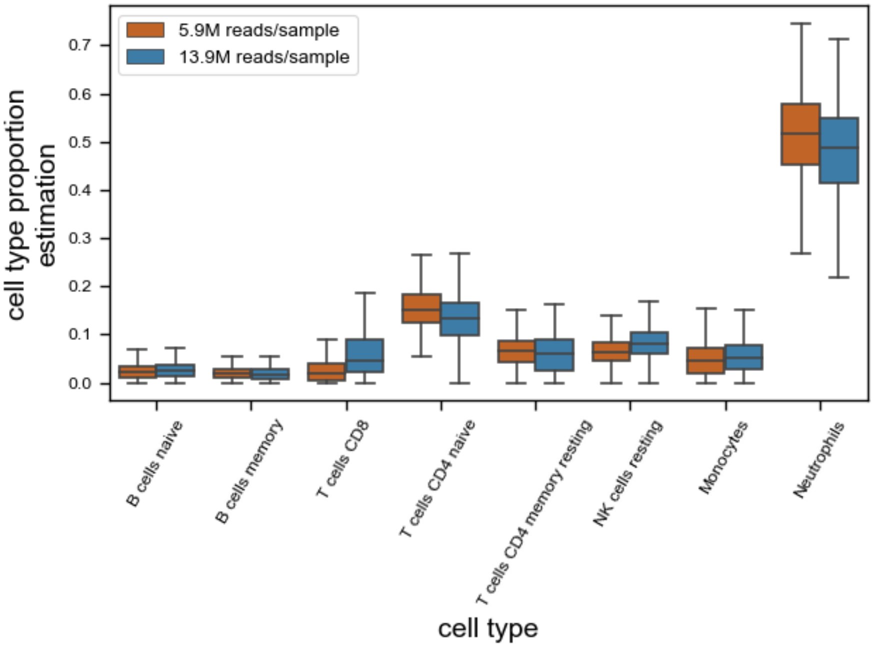


**Figure S11:** *Estimation of cell-type proportions.* **(S11)** In real data, a comparison of estimated cell type proportions from Cibersortx between lower-coverage (5.9M reads/sample) and moderate-coverage (13.9M reads/sample) RNA-Seq data for the eight most common cell types in whole blood tissue.

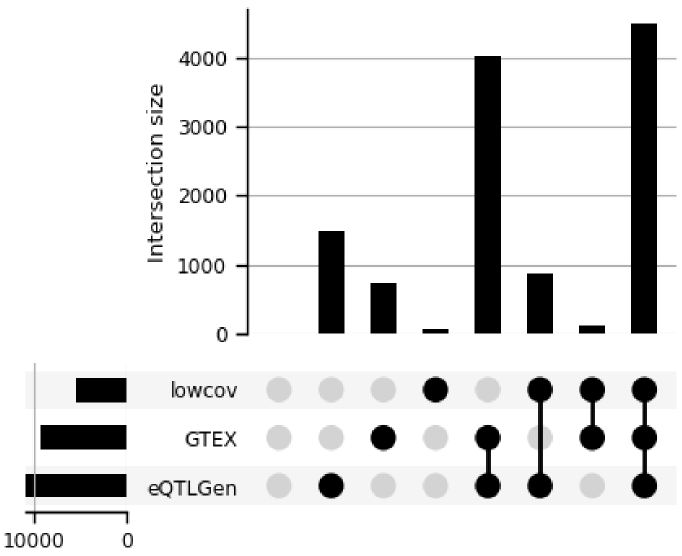


**Figure S12:** *Overlap of significant eGenes using RNA-Seq from three different datasets*. **(S12)** Comparing number of genes with significant associations between three datasets: (1) Lower-coverage RNA-Seq (5.9M reads/sample on average, across 1,496 individuals), (2) GTEX (83M reads/sample on average, across 670 samples), (3) eQTLGen (31,684 individuals, mix of RNA-Seq and MicroArray assays used).

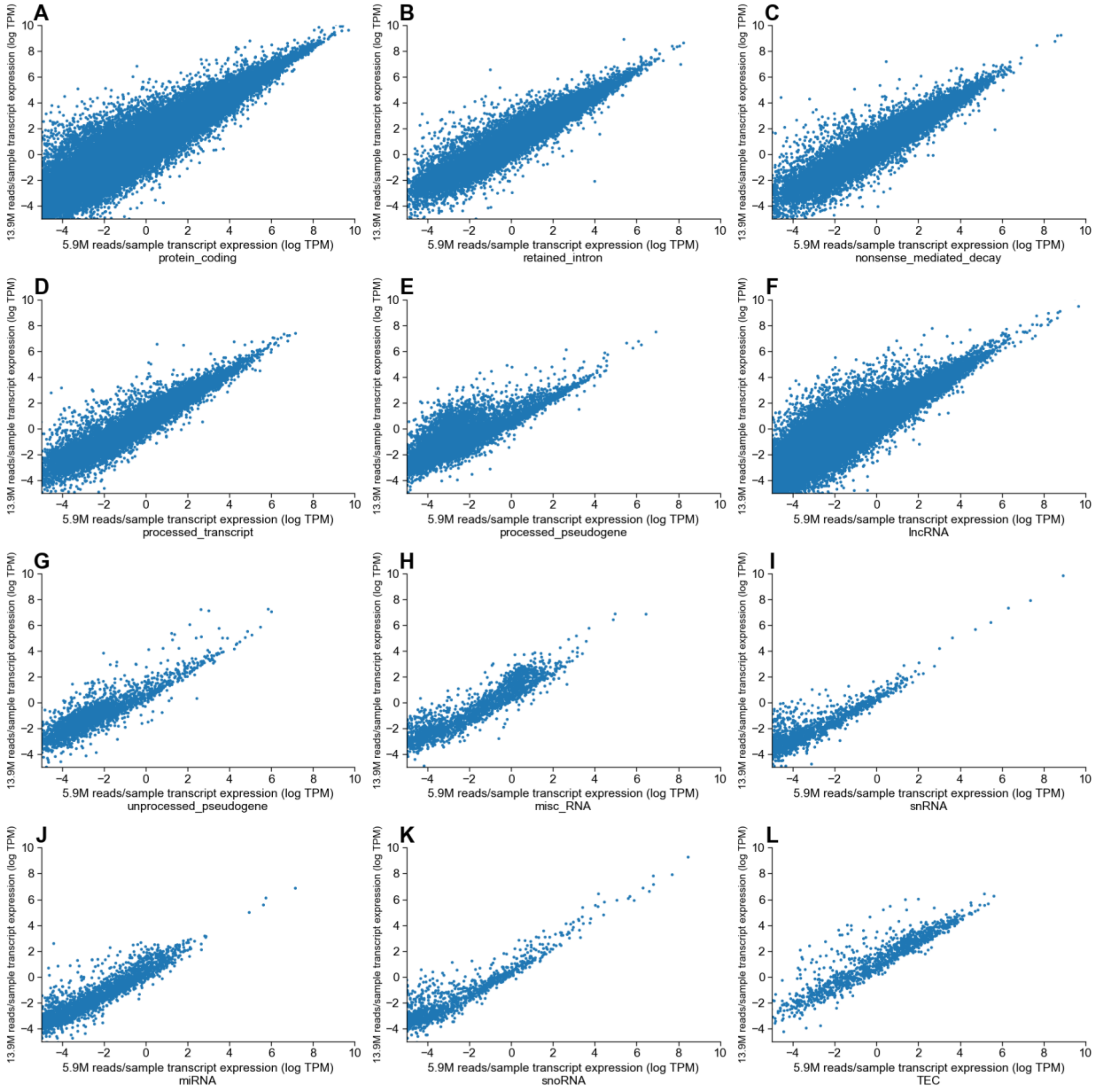


**Figure S13:** *Concordance of transcript expression estimation between lower-coverage RNA-Seq vs moderate-coverage RNA-Seq.* **(S13)** For the 12 most highly represented transcript types (> 1000 transcripts quantified), we show mean expression estimates in lower-coverage RNA-Seq (x-axis) versus moderate-coverage RNA-Seq (y-axis).

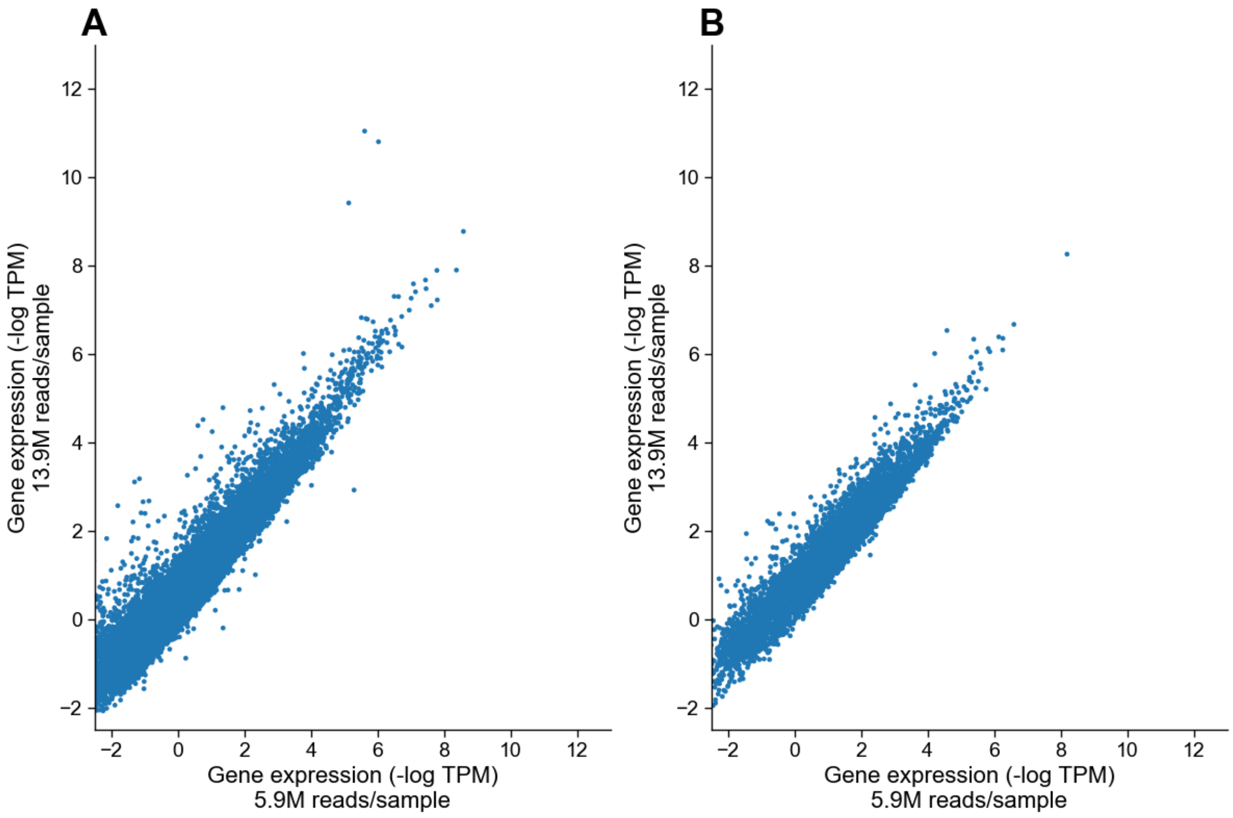


**Figure S14:** *Concordance of gene expression as a function of presence of a predominantly expressed transcript* **(S14A)** Restricting to the 5894 genes with a transcript responsible for at least 50% of the gene's expression in the 13.9M read/sample dataset, comparison of the mean expression (log TPM) across samples, of every gene. $R^2 = 0.91$. **(S14B)** Restricting to the 14711 genes without a transcript responsible for at least 50% of the gene's expression in the 13.9M read/sample dataset, comparison of the mean expression (log TPM) across samples, of every gene. $R^2 = 0.90$.

## SUPPLEMENTARY TABLES

| Platform | N Typed SNPs | N Imputed SNPs |
|---|---|---|
| OmniExpress Exome | 619,690 | 5,576,428 |
| Global Screening Array (GSA) | 514,169 | 4,383,620 |
| COEX | 323,599 | 4,736,265 |
| PsychArray | 378,710 | 4,528,960 |
| Merged | 148,612 | 2,289,732 |

**Table S1:** *Number of typed and imputed SNPs after QC*

| Dataset | Estimated number of total reads mapped in experiment | Estimated cost of experiment | Proportion of cost compared to lower-coverage RNA-Seq experiment | Proportion of eGenes identified compared to lower-coverage RNA-Seq |
|---|---|---|---|---|
| Lower-Coverage or M=5.9M reads/sample (Whole Blood) | ~8.8B | ~$292,000 | 1.0 | 1.0 |
| GTEx | ~55.6B | ~$620,000 | 2.12 | 1.39 |

**Table S2:** *Whole blood RNA-Seq datasets and respective cost estimates*. We describe the lower-coverage and GTEx datasets in terms of estimated cost from our budget model and an estimate for total number of reads used. We assume that the cost of genotyping is $53 per sample (per UCLA Neurogenetics Sequencing Core).

| Transcript type | Number of transcripts | Correlation ($R^2$) of expression |
|---|---|---|
| Protein coding | 83735 | 0.92 |
| Retained intron | 28411 | 0.89 |
| Nonsense mediate decay | 15856 | 0.88 |
| Processed transcript | 14128 | 0.89 |
| Processed pseudogene | 10055 | 0.72 |
| lncRNA | 59133 | 0.85 |
| Unprocessed pseudogene | 2644 | 0.81 |
| miscRNA | 1987 | 0.82 |
| snRNA | 1730 | 0.95 |
| miRNA | 2757 | 0.81 |
| snoRNA | 1367 | 0.94 |
| TEC | 1135 | 0.82 |

**Table S3:** *Transcript-level expression correlations by transcript type.* We quantify expression for 226,390 transcripts. For the 12 transcript types with at least 1000 transcripts represented, we

calculate the correlations between mean transcript expression estimated using lower-coverage RNA-Seq and moderate-coverage RNA-Seq.

**SUPPLEMENTARY NOTE**

Notes about overlap in datasets:

- The samples in the low-coverage whole blood and high-coverage whole blood datasets are completely disjoint – no individuals overlap here.
- 97 individuals have RNA-seq data in both the high-coverage fibroblast dataset and low-coverage whole blood dataset
- 41 individuals have data in the high-coverage fibroblast dataset and the high-coverage whole blood dataset
- In total, 138 individuals overlap between the high-coverage fibroblast RNA-Seq samples and whole blood RNA-Seq samples (low-coverage and high-coverage)
